# Causes of interspecific variation in avian migration distance

**DOI:** 10.1002/ecy.70502

**Published:** 2026-09-16

**Authors:** Neil Paprocki, Courtney J. Conway

**Affiliations:** ^1^ Idaho Cooperative Fish and Wildlife Research Unit, University of Idaho Moscow Idaho USA; ^2^ U.S. Geological Survey, Idaho Cooperative Fish and Wildlife Research Unit, University of Idaho Moscow Idaho USA

**Keywords:** body size, differential migration, flight efficiency, food limitation, phylogenetic comparative methods, thermoregulation

## Abstract

Migration is an awe‐inspiring behavior employed by a diverse suite of taxa throughout the animal kingdom. Avian migration is arguably the most well‐known animal behavior and birds vary widely in how far they migrate, but the ecological mechanisms underlying interspecific variation in migration distance remain unclear. We developed a novel set of predictions deduced from seven mechanistic hypotheses proposed to explain interspecific variation in avian migration distance. We then used a Bayesian phylogenetic comparative analysis to test predictions from these seven hypotheses based on migration data and species traits from 446 species of migratory birds throughout the world based on data from animal‐borne tracking technology. Body mass, seasonal food availability, and morphological/behavioral traits that improve flight efficiency explained significant interspecific variation in migration distance after controlling for absolute breeding latitude (which was positively correlated with migration distance). Specifically, we found migration distance was negatively correlated with body mass and seasonal food availability but positively correlated with hand‐wing index (a measure of wing elongation and pointedness) and soaring flight. Moreover, the negative relationship between body mass and migration distance was not ubiquitous but rather depended on flight mode and intraspecific nonbreeding group size, such that species employing flapping flight during migration and those overwintering in smaller intraspecific groups had the strongest negative relationships between body mass and migration distance. Within group‐living birds, smaller bodied species may gain a foraging or thermoregulatory advantage that shortens migration distances, while larger bodied species may gain an energetic advantage by using flock formations during migration that facilitates longer migrations to more suitable nonbreeding areas. From a life history perspective, maximum annual reproductive investment was negatively correlated with migration distance, while minimum annual reproductive investment and adult annual survival were not correlated with migration distance. Overall, our results support flight efficiency, food limitation, and thermoregulatory‐based hypotheses as causes of interspecific variation in migration distances in birds. Our results also help to refine the numerous and often ambiguous mechanisms underlying the negative relationship between body size and migration distance and refute several hypotheses previously proposed to explain interspecific variation in avian migration distance.

## INTRODUCTION

Migration is an awe‐inspiring phenomenon that has fascinated humans for centuries. Migration can provide ecological connections that link disparate ecosystems via the seasonal movement of organisms across vast geographic scales. Despite our fascination, the underlying causes of variation in migratory behavior are widely debated (Alerstam et al., 2003; Paprocki & Conway, 2025; Soriano‐Redondo et al., 2020). Many interspecific studies attempt to explain why some animals migrate while others do not (Sheard et al., 2020; Soriano‐Redondo et al., 2020), but far fewer explain variation in migration distance among migratory species (Boyle & Conway, 2007; Teitelbaum et al., 2015; Watanabe, 2016). This distinction is important because the ecological mechanisms explaining variation in migration distance and their corresponding life history consequences may be different than those explaining the decision to migrate itself (Boyle & Conway, 2007; Winger & Pegan, 2021).

Migration is arguably more well‐studied in birds than in other taxa, and many prior studies have attempted to explain intraspecific variation in avian migration distance (e.g., Bosman et al., 2012; Hedh & Hedenström, 2020; Macdonald et al., 2016; Paprocki et al., 2026). Intraspecific variation in migration distance is often called differential migration (reviewed in Cristol et al., 1999; Paprocki & Conway, 2025) and at least 12 mechanistic hypotheses have been proposed to explain differential migration in birds (reviewed in Paprocki & Conway, 2025). Interspecific variation in avian migration distance is pronounced but less well studied, and some of the same mechanisms commonly used to explain differential migration could also explain interspecific variation in migration distance (Table 1). A comparative analysis that accounts for phylogenetic relatedness among species is a powerful method for understanding variation in migration more broadly (Boyle & Conway, 2007; Watanabe, 2016). Compared to intraspecific studies, results from a comparative approach enables one to draw more general conclusions about the evolution of migration (Nunn, 2011). Indeed, previous authors have repeatedly advocated for a thorough comparative analysis to better elucidate the mechanisms responsible for interspecific variation in avian migration distance (Cristol et al., 1999; Jahn & Cueto, 2012; Paprocki & Conway, 2025). Despite these pleas, surprisingly few studies have utilized a comparative approach to explain variation in migration distance (but see Boyle & Conway, 2007; Levey & Stiles, 1992; Watanabe, 2016). Comparative analyses and field experiments within the suboscine clade Tyranni and among tropical frugivores reported broad support for food limitation, and adaptations meant to overcome food limitation (e.g., group foraging, dietary specialization), as underlying causes of variation in migration distance (Boyle et al., 2011; Boyle & Conway, 2007). Morphological and behavioral adaptations for improved flight efficiency (e.g., wing morphology, flight mode) are also associated with variation in migration distance across species (Watanabe, 2016). However, a more taxonomically comprehensive comparative analysis that explicitly tests predictions associated with mechanisms underlying a broader suite of hypotheses proposed to explain variation in migration distance is needed to better understand the causes of variation in avian migratory behavior worldwide.

**TABLE 1:** Summary of the mechanistic hypotheses evaluated in this study that may explain interspecific variation in avian migration distances.

Hypothesis	Ecological process	Mechanistic explanation	References
1. Thermal tolerance	Nonbreeding thermoregulatory ability	Larger body size increases tolerance to cold temperatures (via allometrically reduced heat loss because of reduced surface area to volume ratio) which results in shorter migrations	Calder (1974), James (1970), Ketterson and Nolan (1976)
2. Food limitation	Nonbreeding ability to survive food limitation	Species with a greater ability to survive food limitation during the nonbreeding season (via a variety of potential mechanisms) migrate shorter distances	Boyle and Conway (2007), Levey and Stiles (1992)
3. Fasting endurance	Nonbreeding fasting ability	Larger body size increases the ability to fast between meals (via the ability to store greater energy reserves that are metabolized at an allometrically slower rate) which results in shorter migrations because food availability is assumed lower at shorter migration distances	Calder (1974), Ketterson and Nolan (1976)
4. Dietary specialization	Nonbreeding ability to switch diet	Food limitation during the nonbreeding period is caused by differences in diet breadth among species; therefore the ability to switch food types when preferred food is scarce reduces food limitation and shortens migration distances	Boyle et al. (2011)
5. Social dominance	Competition for nonbreeding resources in proximity to breeding grounds	Dominant species with a greater ability to compete for shared resources (usually food) closer to the breeding grounds will exclude subordinate species from accessing resources, forcing subordinates to migrate farther	Fretwell and Lucas (1969), Gauthreaux (1978), Paprocki and Conway (2025)
6. Arrival time	Competition for breeding opportunities	Greater competition for breeding opportunities within species leads to shorter migrations (compared to species with reduced competition for breeding opportunities) because breeding arrival time is positively correlated with migration distance	Ketterson and Nolan (1976), Myers (1981)
7. Flight efficiency	Migration energy expenditure and mortality risk	Variation in flight efficiency during migration allows some species to either migrate faster (time minimization) or exert less energy (energy minimization), reducing mortality risk during migration and allowing some species to migrate farther (to more benign nonbreeding locations)	Alerstam and Lindstrom (1990), Paprocki and Conway (2025)

*Note*: Hypotheses are a combination of those initially proposed to explain intraspecific (thermal tolerance, fasting endurance, social dominance, arrival time) or interspecific variation in migration distance (food limitation, dietary specialization, flight efficiency). Adapted from Paprocki and Conway (2025).

Over the past decade, rapid development and miniaturization of animal‐borne tracking technology (e.g., GPS transmitters, geolocators; Kays et al., 2015; Wilmers et al., 2015) has produced extensive data for conducting comparative analyses of bird migration. The movements of hundreds of bird species from around the globe, many of them migratory, have been documented using recent technological advances in a variety of animal‐borne sensors (McKinnon & Love, 2018; Scarpignato et al., 2023; Watanabe, 2016) and the amount of data is growing exponentially. The surge in avian migration literature derived from animal‐borne tracking technology (hereafter just “tracking technology”) facilitates a rigorous comparative analysis that can help explain underlying causes of variation in migration distance with tremendous spatial resolution and phylogenetic representation. Tracking technology allows for more precise calculation of migration distances while controlling for breeding latitude which is known to covary with migration propensity (Sheard et al., 2020) and distance (Briedis et al., 2016; Wang et al., 2024).

We leveraged information on migration distances to conduct a comparative analysis among 446 bird species throughout the world that controls for phylogenetic relatedness. We tested a novel suite of predictions associated with seven mechanistic hypotheses proposed to explain interspecific variation in avian migration distances (Appendix S1: Table S1). Our analysis included 13 species‐level traits, and we included five interactions to help refine potential mechanisms underlying any observed relationship between body size and migration distance. We also investigated the life history consequences of variation in migration distance and how this aligns with the slow‐fast life history continuum (Winger & Pegan, 2021) to elucidate the likely effects of changing migratory behavior in response to future global change.

## MATERIALS AND METHODS

### Mechanistic hypotheses and predictions

Many hypotheses have been proposed to explain variation in avian migration distance (Paprocki & Conway, 2025). We tested a suite of seven hypotheses (Table 1) that were a combination of older, well‐known hypotheses developed to explain intraspecific variation in migration distance (e.g., social dominance, arrival time, fasting endurance, thermal tolerance; Gauthreaux, 1978; James, 1970; Ketterson & Nolan, 1976; Myers, 1981) and hypotheses that were supported in other comparative analyses explaining interspecific variation in migration distance (e.g., food limitation, dietary specialization, flight efficiency; Boyle & Conway, 2007; Boyle et al., 2011; Levey & Stiles, 1992). We also tested a very general food limitation hypothesis that incorporated mechanisms from as many as seven distinct hypotheses (Paprocki & Conway, 2025) because food limitation has been implicated as a major factor influencing interspecific variation in migration distance (e.g., Boyle & Conway, 2007; Levey & Stiles, 1992). Some hypotheses proposed to explain avian migration distance have partially overlapping mechanisms, and so we selected species traits that underpin the ecological mechanism(s) of at least one, but preferably more than one of the seven hypotheses tested. We then deduced directional predictions based on the ecological mechanism underlying each hypothesis (Appendix S1: Table S1). The explicit list of hypotheses and deduced prediction matrix will be useful for future efforts to test hypotheses explaining variation in migration distance, and a complete justification and explanation of all predictions can be found in Appendix S1: Section S1.

### Migration distance

We compiled migration distances from avian species tracked via geolocators or satellite/GPS transmitters from the published literature. We used the “litsearchr” R package (Grames et al., 2019) to develop a string of keywords (Appendix S1: Section S1) to search Web of Science and Google Scholar for migration studies utilizing tracking technology (last searched in July 2025). We included studies in our analysis using the following criteria: (1) individual(s) completed a one‐way seasonal migration from breeding to nonbreeding or vice versa; (2) straight‐line (i.e., great circle) migration distance and breeding location could be extracted or calculated; and (3) when multiple studies or populations were found for a species, we selected the study or population with the largest sample size. We excluded a relatively small number of species classified as resident (movement distances <100 km; *n* = 15) because prior comparative analyses suggested the mechanisms underlying the decision to migrate may be different than those that influence migration distance (Boyle & Conway, 2007; Winger & Pegan, 2021). We excluded movement distances <100 km because it can be challenging to distinguish them from other nonbreeding movements such as home range expansion, short‐distance dispersal, or extended daily traveling distances to foraging or roosting locations. We used mean straight‐line, population‐level migration distance from direct tracking data as our response variable for several reasons. Straight‐line migration distances from direct tracking data have been used in previous comparative analyses (Watanabe, 2016) and were relatively unbiased to calculate, particularly from published maps displaying individual tracking data where migration distances were otherwise unreported. Conversely, actual travel distances (e.g., migration distance along the path of travel) are more sensitive to location frequency and transmitter type and were more difficult to calculate from published migration maps or were unreported resulting in substantially lower sample sizes (*n* = 171 species). Straight‐line distances (which can underestimate actual distance traveled) have arguably less bias than actual travel distances due to the variation in temporal sampling intervals and spatial measurement error within and among tracking devices (Noonan et al., 2019; Ranacher et al., 2016; Rowcliff et al., 2012). Straight‐line migration distances can also be calculated from species range maps and are therefore theoretically available for all species globally (see Dufour et al., 2020). However, distances derived from range maps are problematic for at least four reasons: (1) the source(s) or data used to generate them are rarely identified and their rigor is not reported; (2) they often include substantial sedentary or partially migratory portions of species' ranges where many individuals do not migrate; (3) they do not identify population‐level connectivity and distances; and (4) they are not intuitively calculated from cosmopolitan species, especially those with breeding ranges that span both northern and southern hemispheres. Population‐level migration distances as we use here also allow for a more precise controlling of breeding latitude, which is known to covary with migration distance (Briedis et al., 2016; Wang et al., 2024). Finally, actual travel distances (Pearson correlation coefficient = 0.93 for 171 species with both straight‐line and actual travel distance from the same breeding population) and aggregated mean distance among intraspecific populations (Pearson correlation coefficient = 0.95 for 161 species with data from multiple populations) were both highly correlated with population‐level, straight‐line migration distances.

### Species traits

We collected data on species traits (Appendix S1: Table S2) from the literature supplemented with information from species experts when no published data could be located. Additional methodological details on the development of species traits can be found in Appendix S1: Section S1. We obtained body mass, bill length (culmen length), and hand‐wing index ($100\times \mathrm{Kip}p^{\prime }s\;\text{distance}÷\text{wing length}$) from Tobias et al. (2022). We categorized territoriality on a scale of 1 to 3 (Sheard et al., 2020; defined in Tobias et al., 2016): 1 = non‐territorial; 2 = seasonally or weakly territorial; or 3 = year‐round territorial. We quantified the percent area of tarsus (i.e., tarsometatarsus and digits) feathering as a metric of thermoregulatory ability based on high‐quality photos from the Cornell Lab of Ornithology's Macaulay Library, supplemented by species experts and examination of museum specimens (Appendix S1: Figure S1). We scored nest substrate availability for each species on a scale of 1 to 5: 1 = species with extremely limited nest substrate availability and 5 = species with ample nest substrate availability. We estimated the proportion of nests for a given species that were functionally secondary cavity nests given that this trait influences other aspects of life history (Martin, 1995): 0 (0% of nests secondary cavity), 0.25, 0.5, 0.75, or 1.0 (100% of nests secondary cavity). We searched species accounts (e.g., Billerman et al., 2022; Cramp, 1977), primary literature, and asked species experts to estimate the degree of nocturnality during the stationary nonbreeding period, where 0 = strictly diurnal and 10 = strictly nocturnal.

We used global eBird data (eBird Basic Dataset, 2024) to calculate a continuous, species‐specific numerical estimate of intraspecific nonbreeding group size during the stationary nonbreeding period. Briefly, we filtered eBird checklists for each species to (1) only include complete checklists submitted during the stationary, nonbreeding months for each species, and (2) filtered checklists to minimize survey effort per checklist (i.e., <30 min and <0.5 km) to maximize the likelihood that each checklist contained as few intraspecific groups as possible. We then calculated the mean checklist count for each species and used this as our estimate of intraspecific nonbreeding group size (which was correlated with previously published binary estimates of group size; Appendix S1: Sections S1 and S2).

We used previously compiled and standardized foraging data from each species (EltonTraits 1.0; Wilman et al., 2014) to calculate novel measures of (1) diet breadth, and (2) seasonal food availability. Diet breadth had the potential to range from 1 to 10: 1 being a highly specialized diet (100% composition from a single diet category) and 10 being a very broad diet (10% composition from each of the 10 diet categories). Seasonal food availability (the relative availability of food on the species' breeding grounds during the nonbreeding season) ranged from 1 to 5: 1 being the lowest availability on the breeding grounds during the nonbreeding season and 5 being the highest availability. Seasonal food availability was calculated using a combination of standardized foraging data from each species (Wilman et al., 2014) and expert scoring of the seasonal availability of seven diet categories averaged among three independent researchers. We used the average expert score for each diet category to calculate seasonal food availability indices for each species because inter‐expert agreement within species was high with an average correlation of 0.89 (Appendix S1: Section S1; Tables S3 and S4).We classified species as either mostly monogamous (polygamy rate <5%) or polygamous (polygamy rate ≥5%; Billerman et al., 2022; Snyder & Creanza, 2019). Flight mode during migration was as a three‐level categorical variable defined using in‐flight videos from the Cornell Lab of Ornithology's Macaulay Library or literature (e.g., Billerman et al., 2022; Cramp, 1977): 1 = obligate flapping (0%–10% soaring); 2 = mix of flapping and soaring (soaring 10%–90% of the time); or 3 = obligate soaring (90%–100% soaring).

We searched species accounts (e.g., Billerman et al., 2022), comparative (Beauchamp, 2022; Bird et al., 2020), and primary literature for estimates of adult annual survival and used the median or estimate that was geographically closest to the breeding location within our database when multiple estimates were found. We used modeled annual survival (Bird et al., 2020) for those species without empirical estimates (*n* = 123 of 446 or 28% of species). We also searched species accounts (e.g., Billerman et al., 2022) and published literature (Myhrvold et al., 2015; Schönwetter & Meise, 1967; Yang et al., 2023) for egg mass, egg length, egg width, clutch size, and number of successful broods per breeding season data to calculate reproductive investment. We used egg length and width data from all species in our dataset to impute missing egg mass values (*n* = 6 species with egg length and width measurements but not mass) using a modeling approach (see Appendix S1: Section S1; Table S11). We then calculated two measures of annual reproductive investment using the following equations: (1) minimum reproductive investment = (egg mass ÷ body mass) × clutch size; (2) maximum reproductive investment = minimum reproductive investment × maximum number of successful broods per breeding season.

### Statistical analyses

Four of our species traits are known to scale allometrically with body mass: bill length, survival, and both minimum and maximum reproductive investment. For these traits, we used residuals from linear models of the log‐transformed species trait (bill length, minimum and maximum reproductive investment) or untransformed species trait (survival) and log‐transformed body mass (Appendix S1: Figures S2–S4). We used residuals from untransformed survival because of improved model fit compared to log‐transformed survival. We log‐transformed migration distance, body mass, and mean nonbreeding group size for all analyses to meet assumptions of normality and facilitate linear relationships among variables. All continuous explanatory and response variables were standardized (mean = 0, SD = 1).

We used Bayesian phylogenetic generalized linear mixed models (PGLMMs) with log‐transformed migration distance as our response variable. We implemented models using the “brms” package (Bürkner, 2017) within the R statistical environment (2025). All models included absolute breeding latitude as a control variable (known to covary with migration distance; Briedis et al., 2016; Wang et al., 2024) and phylogenetic relatedness among species as a random intercept because closely related species with shared evolutionary history may not be independent (Nunn, 2011). We downloaded 50 random phylogenetic trees from the Hackett backbone via birdtree.org (Jetz et al., 2012) to account for phylogenetic relatedness. We trimmed all phylogenies to only include species present within any given model (maximum = 446 species). We calculated a phylogenetic signal (Pagel's lambda; Pagel, 1999) for each model using phylogenetic generalized least squares from the “nlme” package (Pinheiro et al., 2025) and a randomly selected phylogenetic tree. We then used the phylosig function within the “phytools” package (Revell, 2012) because comparative analyses that account for phylogenetic relatedness can reduce model accuracy if models do not have a significant phylogenetic signal (Revell, 2010). We retained phylogenetic relatedness among species as a random intercept in all models because phylogenetic signals were all significant at the *p* = 0.05 level (lambda range = 0.26–0.68; Appendix S1: Table S5–S8).

We utilized an iterative modeling approach such that we initially ran individual models for each prediction deduced from the suite of mechanistic hypotheses (Appendix S1: Tables S1 and S5). Next, we retained and incorporated all main effects with 95% credible intervals that did not overlap zero into a single model. We then added each remaining main effect to determine if parameter estimates changed after accounting for the effects of other, informative predictor variables (Appendix S1: Table S6) and retained all main effects with 95% credible intervals that did not overlap zero (top main effects model). We then added interaction terms one at a time to our top main effects model and retained interactions with 95% credible intervals that did not overlap zero (Appendix S1: Table S7). All models during this iterative approach used the same single, randomly selected phylogenetic tree and included uninformative priors with the following model specifications: two Markov chain Monte Carlo (MCMC) chains of 6000 iterations with a burn‐in period of 2000 and a thinning rate of 1. We used a student's *t*‐distribution (nu = 3, mu = 0, tau = 2.5) for phylogenetic and residual variance model components and a diffuse normal distribution (mean = 0, variance = 15) for all explanatory variables. Models containing different subsets of explanatory variables included slightly different subsets of species; range = 395–446 species due to the presence of missing data (Appendix S1: Table S2). For life history analyses, we built four explicit models that contained absolute breeding latitude and then each of our residual life history variables (corrected for allometry): (1) survival (empirical and modeled estimates); (2) survival (empirical only estimates); (3) minimum reproductive investment; and (4) maximum reproductive investment (Appendix S1: Table S8). We ran all life history and top models for each of 50 randomly selected phylogenetic trees with the following specifications: one MCMC chain of 9000 iterations with a burn‐in period of 5000 and a thinning rate of 5. This resulted in 40,000 posterior samples (1 chain × 800 iterations × 50 trees) from which we drew inferences that accounted for both phylogenetic relatedness and phylogenetic uncertainty. We inspected MCMC sample plots and R‐hat values throughout to assess model convergence. We calculated correlations among explanatory variables using adjusted‐*R* ^2^ from linear models. Correlations and generalized variance inflation factors (GVIFs; Fox & Monette, 1992) among explanatory variables were low (max. *R* ^2^ = 0.356; max. GVIF = 2.48; Appendix S1: Tables S9 and S10). We estimated the overall explanatory power of our predictor variables (which represented mechanisms underlying differential migration distance hypotheses) via marginal *R* ^2^ values calculated from control (absolute breeding latitude only) and top main effects PGLMMs as the proportion of total variance explained by the fixed effects. We used a variance‐partitioning approach adapted for Bayesian PGLMMs, with variance components estimated from posterior draws. We present means ± SD unless noted otherwise.

## RESULTS

**FIGURE 1:**
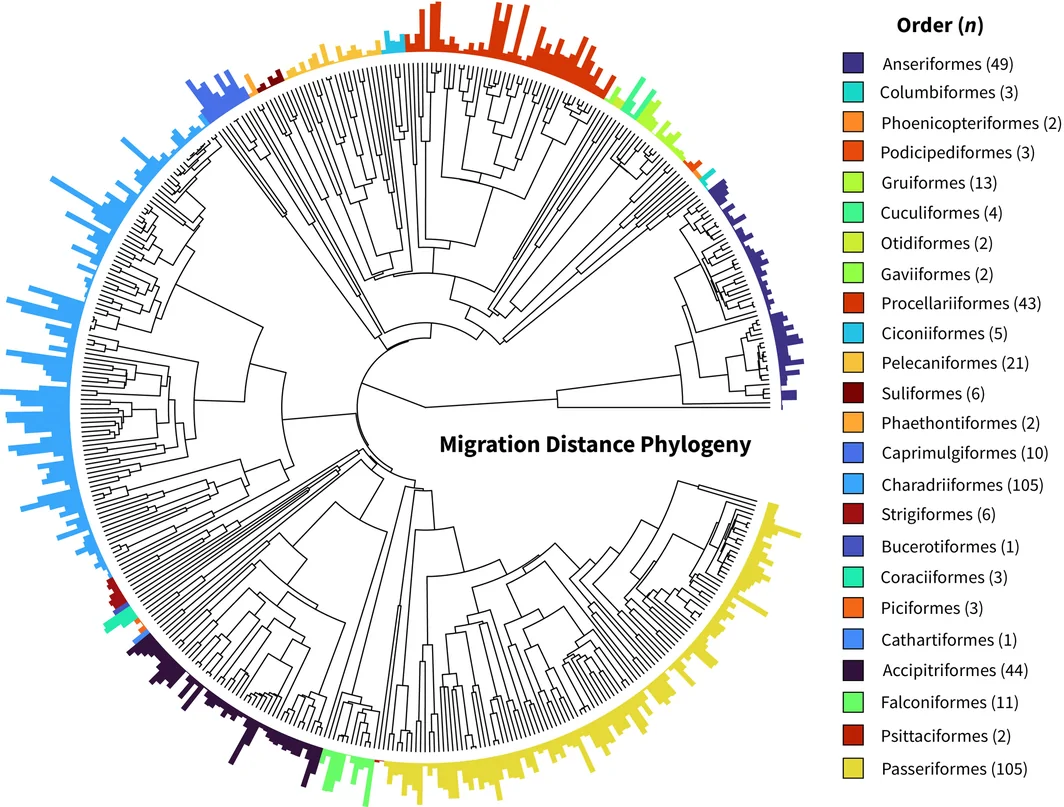
Phylogeny of migration distance across 446 migratory bird species. Migration distance is plotted at the branch tips and colored by phylogenetic order with sample size in parentheses. Orders listed in counterclockwise sequence within the phylogeny starting with Anseriformes.

**FIGURE 2:**
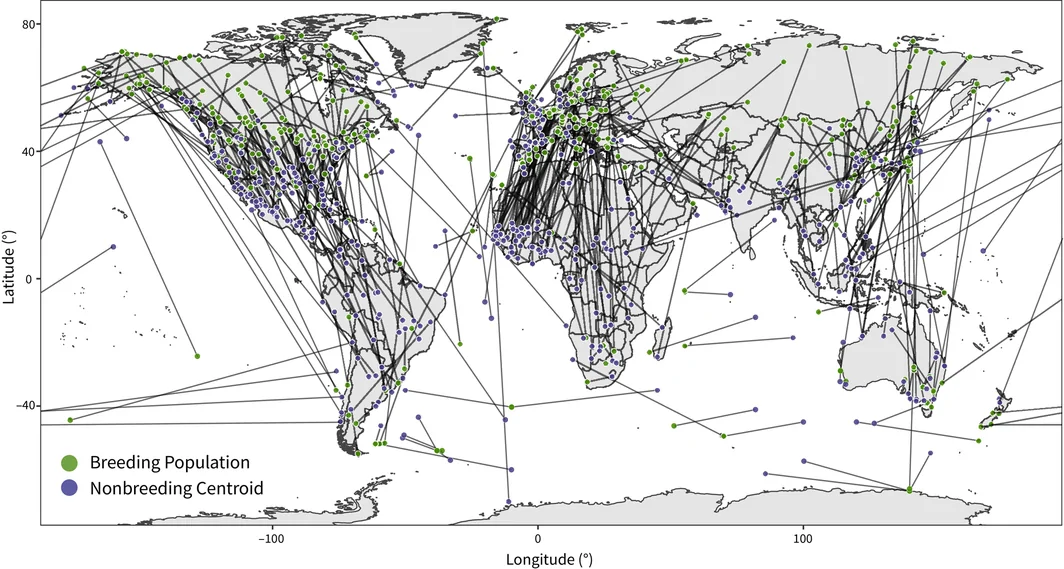
Global map showing mean breeding population locations (green dots) connected by straight lines to mean nonbreeding locations (purple dots) for the 446 species in our comparative analysis to test hypotheses regarding the cause(s) of variation in avian migration distances.

Migration distances were highly variable among the 446 species and ranged from 103 km in trumpeter swan *Cygnus buccinator* to 16,225 km in arctic tern *Sterna paradisaea* (mean distance across the 446 species = 3477 ± 2808 km; Figure 1, Figure 2). Absolute breeding latitude was positively correlated with migration distance (*z* = 0.38, 95% CI = 0.29, 0.46; Appendix S1: Figure S5) and so we included breeding latitude as a control variable in all models.

### Interspecific variation in migration distance: Hypotheses and predictions

We found no support for the arrival time hypothesis, and essentially no support for the dietary specialization hypothesis because the effect of diet breadth on migration distance lessened after controlling for body size (Appendix S1: Section S1; Tables S5 and S6). In contrast, we discovered at least some support for the following five hypotheses: thermal tolerance, food limitation, fasting endurance, social dominance, and flight efficiency (Appendix S1: Table S1). Overall, our top main effects model (absolute breeding latitude + body mass + hand‐wing index + flight mode + seasonal food availability; marginal *R* ^2^ = 0.45) explained 20% more variation than our control model (absolute breeding latitude only; marginal *R* ^2^ = 0.25), indicating that our fixed effects that explicitly represented a priori mechanisms underlying differential migration distance hypotheses contributed substantially to explaining interspecific variation in migration distance.

**FIGURE 3:**
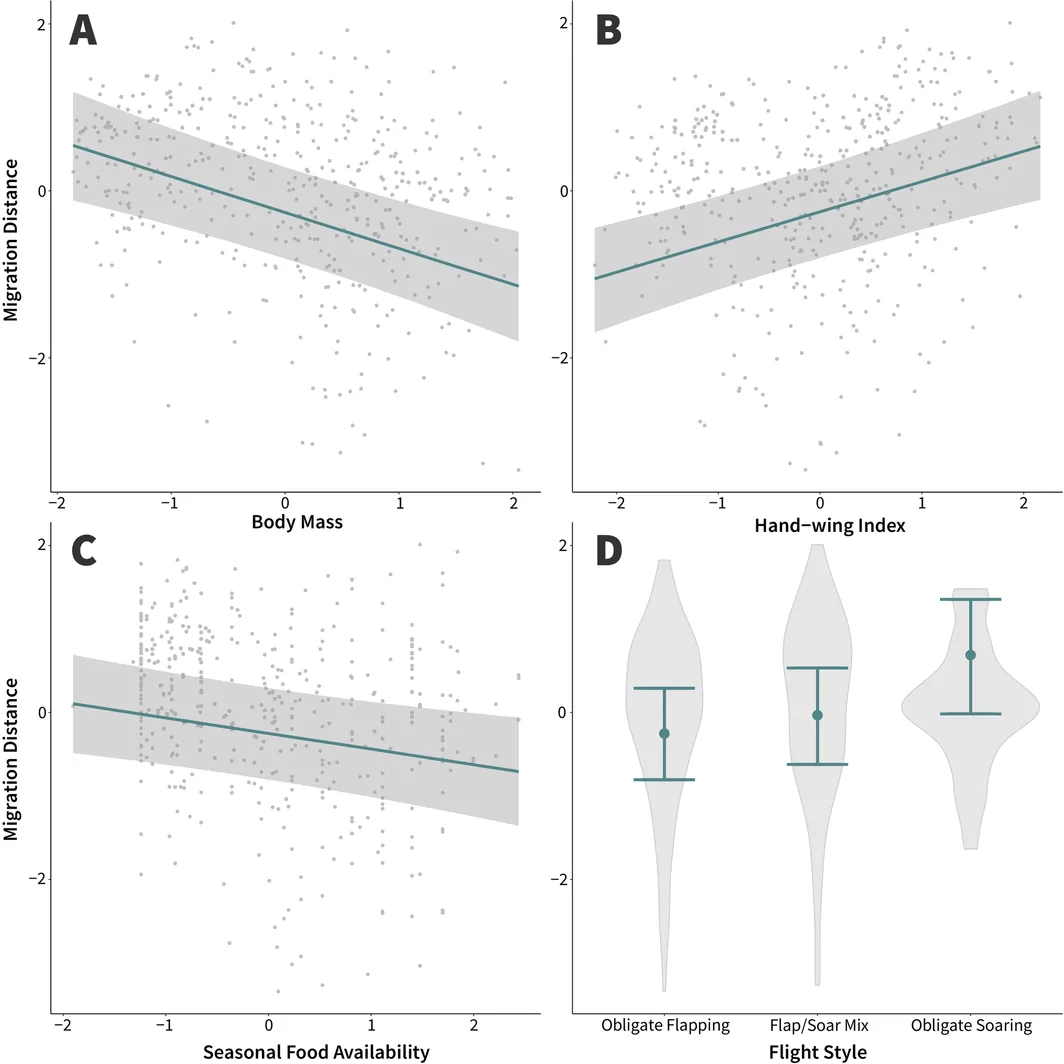
Migration distance was explained by (A) body mass, (B) hand‐wing index, (C) seasonal food availability, and (D) flight mode. Shaded regions around trend lines (A–C) and whiskers (D) represent 95% credible intervals from a Bayesian phylogenetic generalized linear mixed models (PGLMMs) predicting log‐transformed and scaled migration distance including absolute breeding latitude (top distance main effects model). Gray points (1 per species; A–C) or violin plots (D) depict raw data but do not account for the conditional effects of other model variables.

**FIGURE 4:**
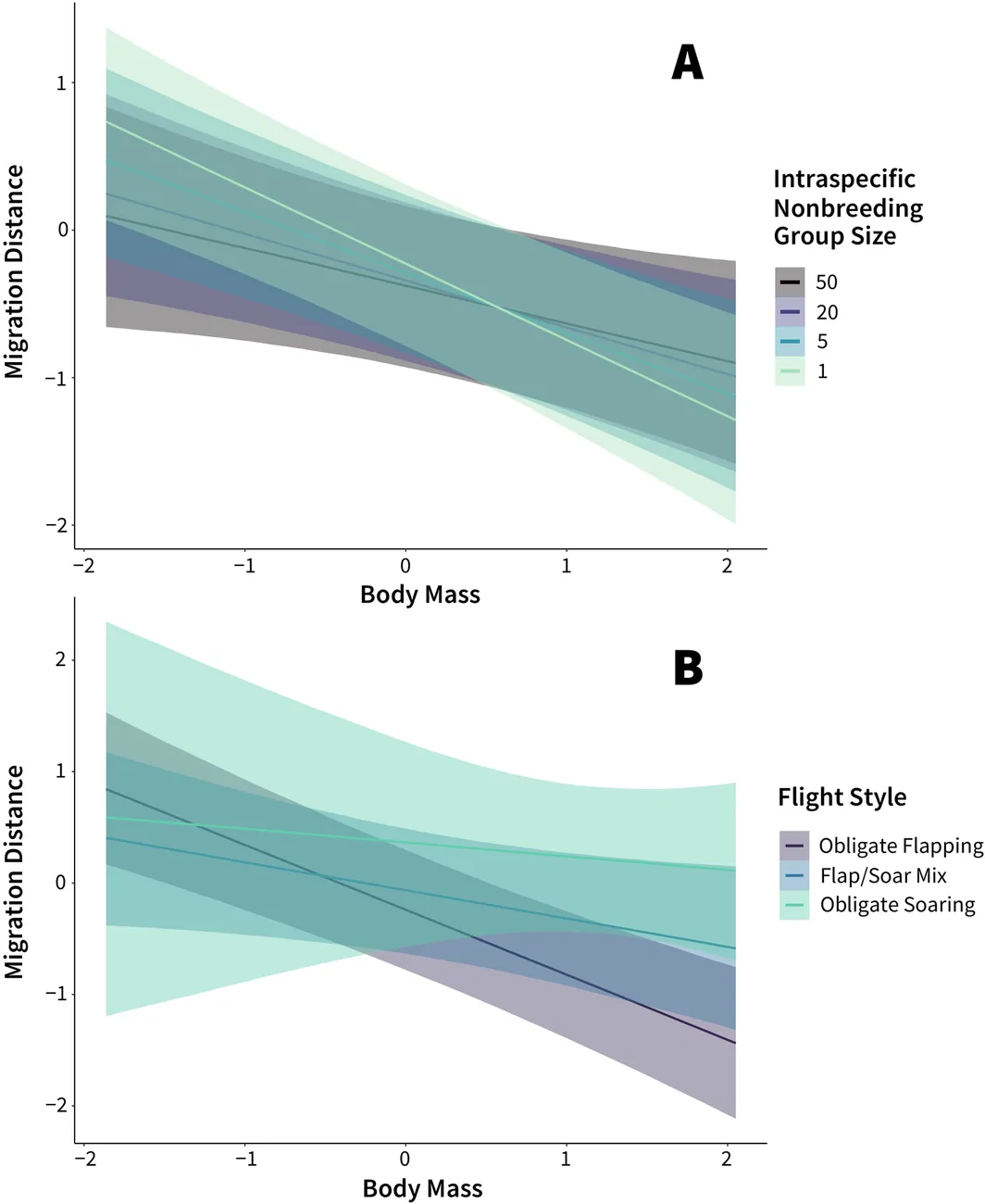
The negative relationship between body mass and migration distance depends on (A) intraspecific nonbreeding group size, and (B) flight mode. Trend lines and shaded regions representing 95% credible intervals are from Bayesian phylogenetic generalized linear mixed models (PGLMMs) predicting log‐transformed and scaled migration distance including all other main effects from our top main effects model.

The thermal tolerance, food limitation, fasting endurance, social dominance, and flight efficiency hypotheses all predicted that body mass should be negatively correlated with migration distance, and that the relationship between body mass and migration distance should depend on intraspecific nonbreeding group size. We found support for both predictions as body mass was negatively correlated with migration distance (*z* = −0.43, 95% CI = −0.61, −0.25; Figure 3A) and the relationship between body mass and migration distance became more negative as intraspecific nonbreeding group sizes decreased (*z* = 0.11, 95% CI = 0.01, 0.20; Figure 4A).

The relationship between body mass and migration distance also depended on flight mode (flap/soar mix *z* = 0.33, 95% CI = 0.04, 0.62; obligate soaring *z* = 0.46, 95% CI = −0.12, 1.04; reference category: obligate flapping) and was most negative in obligate‐flapping migrants (Figure 4B), supporting a prediction specific to the flight efficiency hypothesis. We also documented support for two other predictions specific to the flight efficiency hypothesis: (1) hand‐wing index was positively correlated with migration distance (*z* = 0.36, 95% CI = 0.21, 0.52; Figure 3B), and (2) flight mode was associated with migration distance such that obligate soaring species migrated farther than other flight modes (obligate flapping *z* = −0.94, 95% CI = −1.42, −0.43; flap/soar mix *z* = −0.72, 95% CI = −1.14, −0.28; reference category: obligate soaring; Figure 3D).

Finally, we discovered seasonal food availability was negatively correlated with migration distance (*z* = −0.19, 95% CI = −0.32, −0.06; Figure 3C), supporting a prediction specific to the food limitation hypothesis. All other predictions deduced from the seven hypotheses proposed to explain interspecific variation in migration distance were not supported (i.e., 95% CIs overlapped zero) and included the following: bill length (corrected for body size), extent of tarsus feathering, nest substrate limitation, degree of secondary cavity nesting, polygamy, nocturnality, intraspecific nonbreeding group size, diet breadth, territoriality, body mass × diet breadth, body mass × nocturnality, or body mass × hand‐wing index (Appendix S1: Tables S5–S7).

### Life history consequences of interspecific variation in migration distance

**FIGURE 5:**
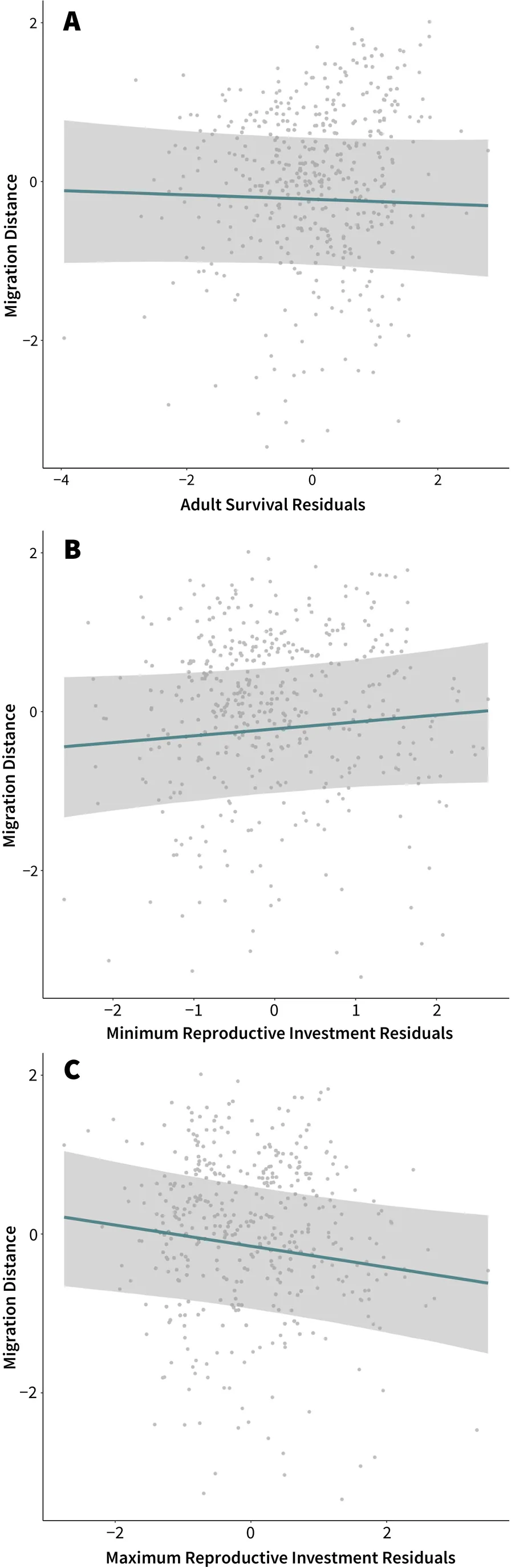
Life history consequences of interspecific variation in migration distance: (A) adult annual survival, (B) minimum and (C) maximum reproductive investment. Trend lines and shaded regions representing 95% credible intervals (only maximum reproductive investment credible intervals did not overlap zero) are from Bayesian phylogenetic generalized linear mixed models (PGLMMs) predicting log‐transformed and scaled migration distance including absolute breeding latitude with life history residuals correcting for allometry. Gray points (1 per species) depict raw data but do not account for the conditional effects of absolute breeding latitude.

After controlling for absolute breeding latitude and body size, survival (*z* = −0.03, 95% CI = −0.14, 0.08; Figure 5A) and minimum reproductive investment (*z* = 0.09, 95% CI = −0.06, 0.23; Figure 5B) were not associated with migration distance while maximum reproductive investment was negatively associated with migration distance (*z* = −0.13, 95% CI = −0.26, −0.01; Figure 5C; Appendix S1: Table S8). The relationship between survival and migration distance was similar when using only empirical (i.e., not modeled; *n* = 323 species) estimates of adult annual survival (*z* = 0.01, 95% CI = −0.10, 0.13; Appendix S1: Table S8).

## DISCUSSION

We used a global, hypothesis‐driven comparative approach to identify the mechanisms underlying interspecific variation in avian migration distances. Of the seven mechanistic hypotheses that we tested to explain interspecific variation in migration distance, our results provided some support for five hypotheses and refuted two hypotheses. We found broad support for flight efficiency, food limitation, and thermoregulation as causes of interspecific variation in migration distance. Importantly, ours is the first study to simultaneously test more than two mechanistic hypotheses explaining interspecific variation in avian migration distances using a comparative approach. Our results corroborate those from several other comparative analyses in that we identified broad support for food limitation (Boyle & Conway, 2007) and flight efficiency hypotheses (Sheard et al., 2020; Watanabe, 2016). But our results did not corroborate previous comparative support for the dietary specialization hypothesis (Boyle et al., 2011).

We reported a strong negative relationship between body mass and migration distance, corroborating both intraspecific (Hegemann et al., 2015; Lundblad & Conway, 2020) and interspecific comparative studies in birds (Wang et al., 2024; Watanabe, 2016). However, a negative relationship between body mass and migration distance has limited utility for inferring causal relationships because six of the seven mechanistic hypotheses we tested make this same prediction. Importantly, our study is the first to demonstrate an interaction between intraspecific nonbreeding group size and body mass and corroborates results from two prior comparative studies that reported an association between group size and migration distance (Beauchamp, 2011; Boyle & Conway, 2007). The nature of the interaction we found indicates that group size is negatively related to migration distance within smaller species, a pattern that was also found within a group of mostly small (<100 g) suboscine birds (Boyle & Conway, 2007). Foraging efficiency appears to be a key factor influencing group living more generally (Beauchamp, 2022), and within smaller species, the negative relationship between nonbreeding group size and migration distance could: (1) improve thermal tolerance in cold weather if foraging groups also roost communally (Beauchamp, 1999; Hay, 1983; thermal tolerance hypothesis), (2) improve the ability to locate or compete for food (food limitation, fasting endurance, or social dominance hypotheses), or (3) improve the ability to forage more efficiently once food is located because of reduced predator vigilance per individual (Beauchamp, 1998, 2010; food limitation or fasting endurance hypotheses). Additional research focused on the mechanistic ambiguity underlying the negative relationship between intraspecific nonbreeding group size and migration distance within smaller bird species is needed to better clarify the mechanism(s) responsible.

In contrast, intraspecific nonbreeding group size was positively related to migration distance within larger species. We can think of only one mechanism that would explain why larger groups migrate farther within larger species (but not smaller species; Larkin & Szafoni, 2008): improved flight efficiency during migration if these same species also migrate in larger flock formations (Beauchamp, 2011). Larger body sizes among all flying animals poses significant constraints on migration in terms of the energetic expenditure needed to fly (Hein et al., 2012; Pennycuick, 2008). Flocking behavior within larger species that utilized tight flock formations (e.g., oblique or V‐shaped formations) can lead to energy savings and more efficient flight (Weimerskirch et al., 2001) perhaps overcoming the flight constraints of larger body sizes and facilitating longer migration distances to more hospitable nonbreeding areas. We did not incorporate group size during migration into our comparative analysis, but many of the larger‐bodied species in our dataset with large nonbreeding group sizes are also known to migrate in V‐shaped group formations (most notably waterfowl species; Billerman et al., 2022). We also found an interaction between body mass and flight mode that suggests the use of soaring flight can also mitigate the energetic flight constraints imposed by a larger body size (Pennycuick, 2008). Our results corroborate a similar finding from another comparative study using a smaller set of migratory birds (Watanabe, 2016) and broadly conforms to bird migration theory because migration speed is positively correlated with body mass in soaring birds (Hedenström & Alerstam, 1998; Pennycuick, 2008). Taken together, these two interactions suggest that flight efficiency during migration places significant constraints on migration distance in larger‐bodied species that attempt to overcome this constraint by migrating in aerodynamically efficient groups or using soaring flight. Additionally, species living in smaller groups or using flapping flight appear to be most affected by the mechanism(s) underlying the negative relationship between body size and migration distance and would be good targets for future research in this area.

The positive correlation between hand‐wing index and migration distance reported in this study provides additional support for the flight efficiency hypothesis as an underlying cause of the interspecific variation in migration distance and corroborates prior comparative studies that reported a positive association between migration propensity and hand‐wing index or other measures of wing pointedness (Lockwood et al., 1998; Sheard et al., 2020). At least two mechanisms may explain why flight efficiency is associated with migration distance: migration time minimization and migration energy minimization (Paprocki & Conway, 2025). Some studies have used time or energy minimization mechanisms to explain patterns in migration speed, migratory refueling, or migration step‐length distances (i.e., distances between stopovers; Alerstam & Hedenström, 1998) but these same mechanisms could influence total migration distance (Hedenström & Alerstam, 1998). However, it is not clear whether greater hand‐wing indices are a cause or consequence of longer migrations (Kennedy et al., 2016; Sheard et al., 2020), a common problem in ecology. Longer hand‐wing indices may have coevolved with migration distance but could have also promoted the evolution of long‐distance migration if resident or short‐distance migrants with longer hand‐wing indices were more predisposed to evolve into long‐distance migrants (Kennedy et al., 2016).

We found that seasonal food availability is negatively correlated with migration distance corroborating prior comparative work on large mammalian herbivores that suggests food limitation is a key factor influencing migration distance (Teitelbaum et al., 2015). In birds, as many as seven mechanistic hypotheses to explain variation in migration rely on food limitation (Paprocki & Conway, 2025). We suggest that the dietary preference hypothesis best explains our observed relationship between seasonal food availability and migration distance whereby certain diet types (but not dietary specialization per se) are less available during the nonbreeding season (e.g., nectar, invertebrates, fruit) which causes longer migration distances for species that rely on those diet types (Elner & Seaman, 2003; Paprocki & Conway, 2025). Past comparative research reported some support for a dietary preference hypothesis in a single clade of birds (the suborder Tyranni) that are primarily insectivores or frugivores (Boyle & Conway, 2007). Our analysis included species representing seven diet types, providing even broader support for the dietary preference hypothesis.

Our results also provide important context regarding the life history (i.e., survival or reproduction) consequences of migration, and how these may vary with the predicted effects of global change on avian migratory behavior. For example, studies commonly report reduced migratory tendencies or distances in response to climate warming (e.g., Heath et al., 2012; Visser et al., 2009), which could result in a faster pace‐of‐life because we documented that migration distance was negatively correlated with maximum reproductive investment. Our results are similar to a recent comparative study of primarily migratory Passeriformes from North America which also found that maximum reproductive investment was negatively correlated with migration distance (Winger & Pegan, 2021). However, our results differed in that we reported no relationship between survival and migration distance (whereas Winger & Pegan, 2021 reported a positive correlation). Winger and Pegan (2021) used a consistent method to estimate adult apparent survival probabilities using standardized capture‐mark‐recapture surveys. In contrast, we used estimates of adult annual survival generated using a variety of techniques, which could have introduced noise to our dataset and impacted our ability to detect patterns between survival and migration distance. Alternatively, the life history consequences of variation in migratory behavior may depend on taxonomy (i.e., consequences may differ among taxonomic groups) or geographic scope and warrants further study.

Our results are based on 446 species for which we located published migration distances generated from direct tracking technology and is the largest and most taxonomically diverse comparative analysis explaining bird migration distances to date encompassing 60% (24 of 40) of extant avian orders globally. However, species and populations whose migrations have been tracked are disproportionately those that breed in the northern hemisphere (*n* = 446; mean breeding latitude = 36.75°N) compared to all other bird species (*n* = 9548; mean breeding latitude = 1.45°N), and especially in North America and Europe (Figure 2; Appendix S1: Section S2). Moreover, the species that were included in this study had larger body sizes (*n* = 446; mean = 988 g; median = 318 g) compared to all other bird species (*n* = 9548; mean = 235 g; median = 34 g; Appendix S1: Section S2; Scarpignato et al., 2023), an unsurprising byproduct of the gradual miniaturization of tracking technology that has occurred (and continues to occur, e.g., Williamson et al., 2024) over the past ~30 years. While these spatial and size biases do not invalidate the patterns documented in this study, future research that includes more small‐bodied, short distance migratory taxa and populations that breed at tropical and southern latitudes will further broaden our understanding of the causes of interspecific variation in migration distance.

Our global comparative analysis produced a rigorous hypothesis and prediction matrix that can be used to generate predictions for future studies and tests of the hypotheses underlying interspecific variation in migration distance. Overall, we identified broad support for flight efficiency, food limitation, and thermoregulatory‐based hypotheses to explain interspecific variation in migration distance, while refuting dietary specialization and breeding arrival time hypotheses. Some intraspecific studies have reported support for the arrival time hypothesis however (e.g., Brown et al., 2021; Woodworth et al., 2016), suggesting that the mechanisms underlying intra‐ versus interspecific variation in migration distance may differ. We also refined our understanding of the mechanistic ambiguity underlying the commonly observed negative correlation between body mass and migration distance in birds by showing that this relationship depends on both intraspecific nonbreeding group size and flight mode. We believe our results provide a significant step forward, and future studies will be able to build upon our results to further clarify the process(es) responsible for extant variation in migration behavior.

## CONFLICT OF INTEREST STATEMENT

The authors declare no conflicts of interest.

## Supporting information


Appendix S1.

## Data Availability

Data and code (Paprocki & Conway, 2026) are available in Dryad at https://doi.org/10.5061/dryad.zw3r228hp.

## References

[ecy70502-bib-0001] Alerstam, T. , and A. Hedenström . 1998. “The Development of Bird Migration Theory.” Journal of Avian Biology 29: 343–369. 10.2307/3677155.

[ecy70502-bib-0002] Alerstam, T. , A. Hedenström , and S. Åkesson . 2003. “Long‐Distance Migration: Evolution and Determinants.” Oikos 103: 247–260. 10.1034/j.1600-0706.2003.12559.x.

[ecy70502-bib-0003] Alerstam, T. , and Å. Lindstrom . 1990. “Optimal Bird Migration: The Relative Importance of Time, Energy, and Safety.” In Bird Migration: Physiology and Ecophysiology, edited by E. Gwinner , 331–351. Berlin, Heidelberg: Springer. 10.1007/978-3-642-74542-3_22.

[ecy70502-bib-0004] Beauchamp, G. 1998. “The Effect of Group Size on Mean Food Intake Rate in Birds.” Biological Reviews 73: 449–472. 10.1017/S0006323198005246.

[ecy70502-bib-0005] Beauchamp, G. 1999. “The Evolution of Communal Roosting in Birds: Origin and Secondary Losses.” Behavioral Ecology 10: 675–687. 10.1093/beheco/10.6.675.

[ecy70502-bib-0006] Beauchamp, G. 2010. “Relaxed Predation Risk Reduces but Does Not Eliminate Sociality in Birds.” Biology Letters 6: 472–474. 10.1098/rsbl.2009.1063. 20106860 PMC2936213

[ecy70502-bib-0007] Beauchamp, G. 2011. “Long‐Distance Migrating Species of Birds Travel in Larger Groups.” Biology Letters 7: 692–694. 10.1098/rsbl.2011.0243. 21525051 PMC3169065

[ecy70502-bib-0008] Beauchamp, G. 2022. “Flocking in Birds Is Associated with Diet, Foraging Substrate, Timing of Activity, and Life History.” Behavioral Ecology and Sociobiology 76: 74. 10.1007/s00265-022-03183-9.

[ecy70502-bib-0009] Billerman, S. M. , B. K. Keeney , P. G. Rodewald , and T. S. Schulenberg , eds. 2022. Birds of the World. Itaca, NY: Cornell Laboratory of Ornithology https://birdsoftheworld.org.

[ecy70502-bib-0010] Bird, J. P. , R. Martin , H. R. Akçakaya , J. Gilroy , I. J. Burfield , S. T. Garnett , A. Symes , J. Taylor , Ç. H. Sekercioglu , and S. H. M. Butchart . 2020. “Generation Lengths of the World's Birds and Their Implications for Extinction Risk.” Conservation Biology 34: 1252–1261. 10.1111/cobi.13486. 32058610

[ecy70502-bib-0011] Bosman, D. S. , H. J. P. Vercruijsse , E. W. M. Stienen , M. Vincx , L. de Neve , and L. Lens . 2012. “Effects of Body Size on Sex‐Related Migration Vary Between Two Closely Related Gull Species with Similar Size Dimorphism.” Ibis 154: 52–60. 10.1111/j.1474-919X.2011.01182.x.

[ecy70502-bib-0012] Boyle, W. A. , and C. J. Conway . 2007. “Why Migrate? A Test of the Evolutionary Precursor Hypothesis.” The American Naturalist 169: 344–359. 10.1086/511335. 17294370

[ecy70502-bib-0013] Boyle, W. A. , C. J. Conway , and J. L. Bronstein . 2011. “Why Do Some, but Not All, Tropical Birds Migrate? A Comparative Study of Diet Breadth and Fruit Preference.” Evolutionary Ecology 25: 219–236. 10.1007/s10682-010-9403-4.

[ecy70502-bib-0014] Briedis, M. , S. Hahn , L. Gustafsson , I. Henshaw , J. Träff , M. Král , and P. Adamík . 2016. “Breeding Latitude Leads to Different Temporal but Not Spatial Organization of the Annual Cycle in a Long‐Distance Migrant.” Journal of Avian Biology 47: 743–748. 10.1111/jav.01002.

[ecy70502-bib-0015] Brown, J. M. , E. E. van Loon , W. Bouten , K. C. J. Camphuysen , L. Lens , W. Müller , C. B. Thaxter , and J. Shamoun‐Baranes . 2021. “Long‐Distance Migrants Vary Migratory Behavior as Much as Short‐Distance Migrants: An Individual‐Level Comparison from a Seabird Species with Diverse Migration Strategies.” Journal of Animal Ecology 90: 1058–1070. 10.1111/1365-2656.13431. 33496020 PMC8247866

[ecy70502-bib-0016] Bürkner, P. C. 2017. “Brms: An R Package for Bayesian Multilevel Models Using StanR Package.” Journal of Statistical Software 80: 1–28. 10.18637/jss.v080.i01.

[ecy70502-bib-0017] Calder, W. A. 1974. “Consequences of Body Size for Avian Energetics.” In Avian Energetics, edited by R. A. Paynter , 86–144. Cambridge, MA: Nuttall Ornithological Club.

[ecy70502-bib-0018] Cramp, S. , ed. 1977. Handbook of the Birds of Europe, the Middle East and North Africa: The Birds of the Western Palearctic. Oxford: Oxford University Press.

[ecy70502-bib-0019] Cristol, D. A. , M. B. Baker , and C. Carbone . 1999. “Differential Migration Revisited: Latitudinal Segregation by Age and Sex Class.” Current Ornithology 15: 33–88. 10.1007/978-1-4757-4901-4_2.

[ecy70502-bib-0020] Dufour, P. , S. Descamps , S. Chantepie , J. Renaud , M. Guéguen , K. Schiffers , W. Thuiller , and S. Lavergne . 2020. “Reconstructing the Geographic and Climatic Origins of Long‐Distance Bird Migrations.” Journal of Biogeography 47: 155–166. 10.1111/jbi.13700.

[ecy70502-bib-0021] eBird Basic Dataset . 2024. Version: EBD_relMay‐2024. Ithaca, NY: Cornell Lab of Ornithology.

[ecy70502-bib-0022] Elner, R. W. , and D. A. Seaman . 2003. “Calidrid Conservation: Unrequited Needs.” Wader Study Group Bulletin 100: 30–34 https://digitalcommons.usf.edu/wsg_bulletin/vol100/iss1/8.

[ecy70502-bib-0023] Fox, J. , and G. Monette . 1992. “Generalized Collinearity Diagnostics.” Journal of the American Statistical Association 87: 178–183. 10.1080/01621459.1992.1047519.

[ecy70502-bib-0024] Fretwell, S. D. , and H. L. Lucas, Jr. 1969. “On Territorial Behavior and Other Factors Influencing the Habitat Distribution in Birds. I. Theoretical Development.” Acta Biotheoretica 19: 16–36.

[ecy70502-bib-0025] Gauthreaux, S. A. 1978. “The Ecological Significance of Behavioural Dominance.” In Perspectives in Ethology, Vol. 3, edited by P. P. G. Bateson and P. H. Klopfer , 17–54. New York: Plenum Press.

[ecy70502-bib-0026] Grames, E. M. , A. N. Stillman , and M. W. Tingley . 2019. “An Automated Approach to Identifying Search Terms for Systematic Reviews Using Keyword Co‐Occurrence Networks.” Methods in Ecology and Evolution 10: 1645–1654. 10.1111/2041-210X.13268.

[ecy70502-bib-0027] Hay, D. B. 1983. “Physiological and Behavioral Ecology of Communally Roosting Pygmy Nuthatch (*Sitta pygmaea*).” Phd diss, Northern Arizona University.

[ecy70502-bib-0028] Heath, J. A. , K. Steenhof , and M. A. Foster . 2012. “Shorter Migration Distances Associated with Higher Winter Temperatures Suggest a Mechanism for Advancing Nesting Phenology of American Kestrels *Falco sparverius* .” Journal of Avian Biology 43: 376–384. 10.1111/j.1600-048X.2012.05595.x.

[ecy70502-bib-0029] Hedenström, A. , and T. Alerstam . 1998. “How Fast Can Birds Migrate?” Journal of Avian Biology 29: 424–432. 10.2307/3677161.

[ecy70502-bib-0030] Hedh, L. , and A. Hedenström . 2020. “The Migration of a Monogamous Shorebird Challenges Existing Hypotheses Explaining the Evolution of Differential Migration.” Journal of Theoretical Biology 487: 110111. 10.1016/j.jtbi.2019.110111. 31836506

[ecy70502-bib-0031] Hegemann, A. , P. P. Marra , and B. I. Tieleman . 2015. “Causes and Consequences of Partial Migration in a Passerine Bird.” The American Naturalist 186: 531–546. 10.1086/682667. 26655576

[ecy70502-bib-0032] Hein, A. W. , C. Hou , and J. F. Gillooly . 2012. “Energetic and Biomechanical Constraints on Animal Migration Distance.” Ecology Letters 15: 104–110. 10.1111/j.1461-0248.2011.01714.x. 22093885

[ecy70502-bib-0033] Jahn, A. E. , and V. R. Cueto . 2012. “The Potential for Comparative Research Across New World Bird Migration Systems.” Journal of Ornithology 153: 199–205. 10.1007/s10336-012-0849-8.

[ecy70502-bib-0034] James, F. C. 1970. “Geographic Size Variation in Birds and Its Relationship to Climate.” Ecology 51: 365–390. 10.2307/1935374.

[ecy70502-bib-0035] Jetz, W. , G. H. Thomas , J. B. Joy , K. Hartmann , and A. O. Mooers . 2012. “The Global Diversity of Birds in Space and Time.” Nature 491: 444–448. 10.1038/nature11631. 23123857

[ecy70502-bib-0036] Kays, R. , M. C. Crofoot , W. Jetz , and M. Wikelski . 2015. “Terrestrial Animal Tracking as an Eye on Life and Planet.” Science 348: aaa2478. 10.1126/science.aaa2478. 26068858

[ecy70502-bib-0037] Kennedy, J. D. , M. K. Borregaard , K. A. Jønsson , P. Z. Marki , J. Fjeldså , and C. Rahbek . 2016. “The Influence of Wing Morphology Upon the Dispersal, Geographical Distributions and Diversification of the Corvides (Aves; Passeriformes).” Proceedings of the Royal Society B: Biological Sciences 283: 20161922. 10.1098/rspb.2016.1922. PMC5204152 27974521

[ecy70502-bib-0038] Ketterson, E. D. , and V. Nolan, Jr. 1976. “Geographic Variation and Its Climatic Correlates in the Sex Ratio of Eastern‐Wintering Dark‐Eyed Juncos (*Junco hyemalis hyemalis*).” Ecology 57: 679–693. 10.2307/1936182.

[ecy70502-bib-0039] Larkin, R. P. , and R. E. Szafoni . 2008. “Evidence for Widely Dispersed Birds Migrating Together at Night.” Integrative and Comparative Biology 48: 40–49. 10.1093/icb/icn038. 21669771

[ecy70502-bib-0040] Levey, D. J. , and F. G. Stiles . 1992. “Evolutionary Precursors of Long‐Distance Migration: Resource Availability and Movement Patterns in Neotropical Landbirds.” The American Naturalist 140: 447–476. 10.1086/285421.

[ecy70502-bib-0041] Lockwood, R. , J. P. Swaddle , and J. M. V. Rayner . 1998. “Avian Wingtip Shape Reconsidered: Wingtip Shape Indices and Morphological Adaptations to Migration.” Journal of Avian Biology 29: 273–292. 10.2307/3677110.

[ecy70502-bib-0042] Lundblad, C. G. , and C. J. Conway . 2020. “Testing Four Hypotheses to Explain Partial Migration: Balancing Reproductive Benefits with Limits to Fasting Endurance.” Behavioral Ecology and Sociobiology 74: 1–16. 10.1007/s00265-019-2796-3.

[ecy70502-bib-0043] Macdonald, C. A. , E. A. Mckinnon , H. G. Gilchrist , and O. P. Love . 2016. “Cold Tolerance, and Not Earlier Arrival on Breeding Grounds, Explains Why Males Winter Further North in an Arctic‐Breeding Songbird.” Journal of Avian Biology 47: 7–15. 10.1111/jav.00689.

[ecy70502-bib-0044] Martin, T. E. 1995. “Avian Life History Evolution in Relation to Nest Sites, Nest Predation, and Food.” Ecological Monographs 65: 101–127. 10.2307/2937160.

[ecy70502-bib-0045] McKinnon, E. A. , and O. P. Love . 2018. “Ten Years Tracking the Migrations of Small Landbirds: Lessons Learned in the Golden Age of Bio‐Logging.” The Auk: Ornithological Advances 135: 834–856. 10.1642/AUK-17-202.1.

[ecy70502-bib-0046] Myers, J. P. 1981. “A Test of Three Hypotheses for Latitudinal Segregation of the Sexes in Wintering Birds.” Canadian Journal of Zoology 59: 1527–1534. 10.1139/z81-207.

[ecy70502-bib-0047] Myhrvold, N. P. , E. Baldridge , B. Chan , D. Sivam , D. L. Freeman , and S. K. M. Ernest . 2015. “An Amniote Life‐History Database to Perform Comparative Analyses with Birds, Mammals, and Reptiles.” Ecology 96: 3109. 10.1890/15-0846R.1.

[ecy70502-bib-0048] Noonan, M. J. , C. H. Fleming , T. S. Akre , J. Drescher‐Lehman , E. Gurarie , A. L. Harrison , R. Kays , and J. M. Calabrese . 2019. “Scale‐Insensitive Estimation of Speed and Distance Traveled from Animal Tracking Data.” Movement Ecology 7: 35. 10.1186/s40462-019-0177-1. 31788314 PMC6858693

[ecy70502-bib-0049] Nunn, C. L. 2011. The Comparative Approach in Evolutionary Anthropology and Biology. Chicago: University of Chicago Press.

[ecy70502-bib-0050] Pagel, M. 1999. “Inferring the Historical Patterns of Biological Evolution.” Nature 401: 877–884. 10.1038/44766. 10553904

[ecy70502-bib-0051] Paprocki, N. , and C. J. Conway . 2025. “The Underlying Causes of Differential Migration: Assumptions, Hypotheses, and Predictions.” Biological Reviews 100: 764–789. 10.1111/brv.13160. 39522953

[ecy70502-bib-0052] Paprocki, N. , and C. J. Conway . 2026. “Data and Codes from: Causes of Interspecific Variation in Avian Migration Distance [Dataset].” Dryad Digital Repository. 10.5061/dryad.zw3r228hp.42750000

[ecy70502-bib-0053] Paprocki, N. , J. Kidd , R. Warne , A. Macedo , and C. J. Conway . 2026. “Causes of Differential Migration Distance: Test of Seven Mechanistic Hypotheses in an Arctic Raptor.” Behavioral Ecology 36: araf072. 10.1093/beheco/araf072.

[ecy70502-bib-0054] Pennycuick, C. J. 2008. Modelling the Flying Bird. Amsterdam: Elsevier.

[ecy70502-bib-0077] Pinheiro, J. , D. Bates , and R Core Team . 2025. “nlme: Linear and Nonlinear Mixed Effects Models.” R package version 3.1‐168. 10.32614/CRAN.package.nlme.

[ecy70502-bib-0055] Ranacher, P. , R. Brunauer , W. Trutschnig , S. van der Spek , and S. Reich . 2016. “Why GPS Makes Distance Bigger Than They Are.” International Journal of Geographical Information Science 30: 316–333. 10.1080/13658816.2015.1086924. 27019610 PMC4786863

[ecy70502-bib-0056] Revell, L. J. 2010. “Phylogenetic Signal and Linear Regression on Species Data.” Methods in Ecology and Evolution 1: 319–329. 10.1111/j.2041-210X.2010.00044.x.

[ecy70502-bib-0057] Revell, L. J. 2012. “Phytools: An R Package for Phylogenetic Comparative Biology (and Other Things).” Methods in Ecology and Evolution 3: 217–223. 10.1111/j.2041-210X.2011.00169.x.

[ecy70502-bib-0058] Rowcliff, J. M. , C. Carbone , R. Kays , B. Kranstauber , and P. A. Jansen . 2012. “Bias in Estimating Animal Travel Distance: The Effect of Sampling Frequency.” Methods in Ecology and Evolution 3: 653–662. 10.1111/j.2041-210X.2012.00197.x.

[ecy70502-bib-0059] Scarpignato, A. L. , A. E. Huysman , M. F. Jimenez , C. J. Witko , A.‐L. Harrison , N. E. Seavy , M. A. Smith , J. L. Deppe , C. B. Wilsey , and P. P. Marra . 2023. “Shortfalls in Tracking Data Available to Inform North American Migratory Bird Conservation.” Biological Conservation 286: 110224. 10.1016/j.biocon.2023.110224.

[ecy70502-bib-0060] Schönwetter, M. , and W. Meise . 1967. Handbuch der oologie, Vol. 1. Berlin: Akademie‐Verlag.

[ecy70502-bib-0061] Sheard, C. , M. H. C. Neate‐Clegg , N. Alioravainen , S. E. I. Jones , C. Vincent , H. E. A. Macgregor , T. P. Bregman , S. Claramunt , and J. A. Tobias . 2020. “Ecological Drivers of Global Gradients in Avian Dispersal Inferred from Wing Morphology.” Nature Communications 11: 2463. 10.1038/s41467-020-16313-6. PMC7235233 32424113

[ecy70502-bib-0062] Snyder, K. T. , and N. Creanza . 2019. “Polygyny Is Linked to Accelerated Birdsong Evolution but Not to Larger Song Repertoires.” Nature Communications 10: 884. 10.1038/s41467-019-08621-3. PMC6385279 30792389

[ecy70502-bib-0063] Soriano‐Redondo, A. , J. S. Gutiérrez , D. Hodgson , and S. Bearhop . 2020. “Migrant Birds and Mammals Live Faster Than Residents.” Nature Communications 11: 5719. 10.1038/s41467-020-19256-0. PMC7673136 33203869

[ecy70502-bib-0064] Teitelbaum, C. S. , W. F. Fagan , C. H. Fleming , G. Dressler , J. M. Calabrese , P. Leimgruber , and T. Mueller . 2015. “How Far to Go? Determinants of Migration Distance in Land Mammals.” Ecology Letters 18: 545–552. 10.1111/ele.12435. 25865946

[ecy70502-bib-0065] Tobias, J. A. , C. Sheard , A. L. Pigot , A. J. M. Devenish , J. Yang , F. Sayol , M. H. C. Neat‐Clegg , et al. 2022. “AVONET: Morphological, Ecological and Geographical Data for all Birds.” Ecology Letters 25: 581–597. 10.1111/ele.13898. 35199922

[ecy70502-bib-0066] Tobias, J. A. , C. Sheard , N. Seddon , A. Meade , A. J. Cotton , and S. Nakagawa . 2016. “Territoriality, Social Bonds, and the Evolution of Communal Signaling in Birds.” Frontiers in Ecology and Evolution 4: 74. 10.3389/fevo.2016.00074.

[ecy70502-bib-0067] Visser, M. E. , A. C. Perdeck , J. H. van Balen , and C. Both . 2009. “Climate Change Leads to Decreasing Bird Migration Distances.” Global Change Biology 15: 1859–1865. 10.1111/j.1365-2486.2009.01865.x.

[ecy70502-bib-0068] Wang, X. , M. Somveille , A. M. Dokter , W. Cao , C. Cheng , J. Liu , and Z. Ma . 2024. “Macro‐Scale Relationship Between Body Mass and Timing of Bird Migration.” Nature Communications 15: 4111. 10.1038/s41467-024-48248-7. PMC11096376 38750018

[ecy70502-bib-0069] Watanabe, Y. Y. 2016. “Flight Mode Affects Allometry of Migration Range in Birds.” Ecology Letters 19: 907–914. 10.1111/ele.12627. 27305867

[ecy70502-bib-0070] Weimerskirch, H. , J. Martin , Y. Clerquin , P. Alexandre , and S. Jiraskova . 2001. “Energy Saving in Flight Formation.” Nature 413: 697–698. 10.1038/35099670. 11607019

[ecy70502-bib-0071] Williamson, J. L. , E. F. Gyllenhaal , S. M. Bauernfeind , E. Bautista , M. J. Baumann , C. R. Gadek , P. P. Marra , et al. 2024. “Extreme Elevational Migration Spurred Cryptic Speciation in Giant Hummingbirds.” Proceedings of the National Academy of Sciences 121: e2313599121. 10.1073/pnas.2313599121. PMC11126955 38739790

[ecy70502-bib-0072] Wilman, H. , J. Belmaker , J. Simpson , C. de la Rosa , M. M. Rivadeneira , and W. Jetz . 2014. “EltonTraits 1.0: Species‐Level Foraging Attributes of the World's Birds and Mammals.” Ecology 95: 2027. 10.1890/13-1917.1.

[ecy70502-bib-0073] Wilmers, C. C. , B. Nickel , C. M. Bryce , J. A. Smith , R. E. Wheat , and V. Yovovich . 2015. “The Golden Age of Bio‐Logging: How Animal‐Borne Sensors Are Advancing the Frontiers of Ecology.” Ecology 96: 1741–1753. 10.1890/14-1401.1. 26378296

[ecy70502-bib-0074] Winger, B. M. , and T. M. Pegan . 2021. “Migration Distance Is a Fundamental Axis of the Slow‐Fast Continuum of Life History in Boreal Birds.” Ornithology 138: 1–18. 10.1093/ornithology/ukab043.

[ecy70502-bib-0075] Woodworth, B. K. , A. E. M. Newman , S. P. Turbek , B. C. Dossman , K. A. Hobson , L. I. Wassenaar , G. W. Mitchell , N. T. Wheelwright , and D. R. Norris . 2016. “Differential Migration and the Link Between Winter Latitude, Timing of Migration, and Breeding in a Songbird.” Oecologia 181: 413–422. 10.1007/s00442-015-3527-8. 26888571

[ecy70502-bib-0076] Yang, M. , C. T. Callaghan , and J. Wu . 2023. “How Do Birds with Different Traits Respond to Urbanization? A Phylogenetically Controlled Analysis Based on Citizen Science Data and a Diverse Urbanization Measurement.” Landscape and Urban Planning 237: 104801. 10.1016/j.landurbplan.2023.104801.

