# Supplementary material for: Causes of interspecific variation in avian migration distance
Source: Ecology. 2026 Sep 16;107(9):e70502. doi: 10.1002/ecy.70502 (PMC13582435; doi:10.1002/ecy.70502)
Supplement: Supplementary file 1 [file ECY-107-e70502-s001.pdf]

Appendix S1

Causes of interspecific variation in avian migration distance

Neil Paprocki, Courtney Conway

Ecology

Any use of trade, firm, or product names is for descriptive purposes only and does not imply endorsement by the U.S. Government.

Section S1: Supplemental Methods

1. Migration Distance Prediction Justification and Explanation

We present complete justifications and explanations for all deduced predictions (with corresponding hypotheses in parentheses) found in Table S1 by species trait or interaction. We want to acknowledge that we primarily tested the social dominance distance minimization hypothesis (Paprocki & Conway 2025). We also acknowledge that we generally did not distinguish between migration time or energy minimization mechanisms in our testing of the flight efficiency hypothesis (Paprocki & Conway 2025).

Bill length: Bill length (corrected for body size) should be negatively correlated with thermoregulatory ability (Allen's Rule; Symonds & Tattersall 2010); and should therefore be positively correlated with migration distance (thermal tolerance). Bill length (corrected for body size) could be positively correlated with competitive ability (Leighton et al. 2022; if bills are used as weapons to compete for resources during the nonbreeding period); and could therefore be negatively correlated with migration distance (social dominance). Bill length (corrected for body size) could be negatively correlated with flight efficiency (if longer/larger bills increase drag

during flight); and could therefore be negatively correlated with migration distance (flight efficiency). Bill length (corrected for body size) should not be correlated with ability to survive food limitation, ability to fast between meals, ability to switch diets, or competition for breeding opportunities; and should therefore not be correlated with migration distance (food limitation, fasting endurance, dietary specialization, arrival time).

Body mass: Body mass should be positively correlated with thermoregulatory ability (James 1970, Calder 1974), ability to survive food limitation, ability to fast between meals (Calder 1974, Ketterson & Nolan 1976), and ability to compete for resources (Gauthreaux 1978); and should therefore be negatively correlated with migration distance (thermal tolerance, food limitation, fasting endurance, social dominance). Body mass could be positively correlated with ability to switch diet (if larger body size facilitates a greater range of consumable food sizes; Schoener 1967, McNab 1971); and could therefore be negatively correlated with migration distance (dietary specialization). Body mass should be negatively correlated with flight efficiency (especially in species using predominantly flapping flight; Hedenström & Alerstam 1998, Pennycuick 2008); and should therefore be negatively correlated with migration distance within species using predominantly flapping flight. Body mass should not be correlated with competition for breeding opportunities; and should therefore not be correlated with migration distance (arrival time).

Diet breadth: Diet breadth should be positively correlated with the ability to survive food limitation and the ability to switch diets (Boyle *et al*. 2011); and should therefore be negatively correlated with migration distance (food limitation, dietary specialization). Diet breadth could be positively correlated with energy efficiency during migration (if a broader diet improves the ability to refuel during migratory stopovers); and could therefore be positively correlated with

migration distance (flight efficiency). Diet breadth should not be correlated with the ability to thermoregulate, ability to fast between meals, ability to compete for resources, or competition for breeding opportunities; and should therefore not be correlated with migration distance (thermal tolerance, fasting endurance, social dominance, arrival time).

Seasonal food availability: Seasonal food availability should be positively correlated with the ability to survive food limitation (because variation in dietary preferences causes variation in seasonal food availability; Elner & Seaman 2003); and should therefore be negatively correlated with migration distance (food limitation). Seasonal food availability should not be correlated with ability to thermoregulate, ability to fast between meals, ability to switch diet, ability to compete for resources, competition for breeding opportunities, or flight efficiency; and should therefore not be correlated with migration distance (thermal tolerance, fasting endurance, dietary specialization, social dominance, arrival time, flight efficiency).

Flight mode: The propensity for soaring fight should be positively correlated with flight efficiency (Pennycuick 2008); and should therefore be positively correlated with migration distance (flight efficiency). The propensity for soaring flight (i.e., flight mode from flapping to soaring) should not be correlated with ability to thermoregulate, ability to survive food limitation, ability to fast between meals, ability to switch diet, ability to compete for resources, or competition for breeding opportunities; and should therefore not be correlated with migration distance (thermal tolerance, food limitation, fasting endurance, dietary specialization, social dominance, arrival time).

Hand-wing index: Hand-wing index should be positively correlated with flight efficiency (Lockwood et al. 1998, Sheard et al. 2020); and should therefore be positively correlated with migration distance (flight efficiency). Hand-wing index should not be correlated with ability to

thermoregulate, ability to survive food limitation, ability to fast between meals, ability to switch diet, ability to compete for resources, or competition for breeding opportunities; and should therefore not be correlated with migration distance (thermal tolerance, food limitation, fasting endurance, dietary specialization, social dominance, arrival time).

Nest substrate availability: Nest substrate availability should be negatively correlated with competition for breeding opportunities; and should therefore be positively correlated with migration distance (arrival time). Nest substrate availability should not be correlated with ability to thermoregulate, ability to survive food limitation, ability to fast between meals, ability to switch diet, ability to compete for resources, or flight efficiency; and should therefore not be correlated with migration distance (thermal tolerance, food limitation, fasting endurance, dietary specialization, social dominance, flight efficiency).

Secondary cavity use: Secondary cavity use should be positively correlated with competition for breeding opportunities; and should therefore be positively correlated with migration distance (arrival time). Secondary cavity use should be positively correlated with the ability to thermoregulate (if secondary cavity nesters also use cavities for winter roosting offering an improved ability to thermoregulate via behavioral adaptation); and should therefore be negatively correlated with migration distance (thermal tolerance). Secondary cavity use should not be correlated with ability to survive food limitation, ability to fast between meals, ability to switch diet, ability to compete for resources, or flight efficiency; and should therefore not be correlated with migration distance (food limitation, fasting endurance, dietary specialization, social dominance, flight efficiency).

Nocturnality: Degree of nocturnality during the stationary, nonbreeding period could be positively correlated with the ability to survive food limitation via reduced fasting durations

(because birds can actively feed during long winter nights), reduced need to thermoregulate (because birds are active during cold winter nights), or reduced amount of competition for food (because fewer birds are nocturnal; but nocturnality should not be related to fasting *ability*, thermoregulatory *ability*, or competitive *ability*); and should therefore be negatively correlated with migration distance (food limitation). Degree of nocturnality during the stationary, nonbreeding period could be negatively correlated with competition for breeding opportunities (if there is less competition for breeding opportunities among nocturnal species because they are less abundant); and could therefore be positively correlated with migration distance. Degree of nocturnality during the stationary, nonbreeding period should not be correlated with ability to thermoregulate, ability to fast between meals, ability to switch diet, ability to compete for resources, or flight efficiency; and should therefore not be correlated with migration distance (thermal tolerance, fasting endurance, dietary specialization, social dominance, flight efficiency).

Intraspecific nonbreeding group size: Intraspecific nonbreeding group size during the stationary, nonbreeding period should be positively correlated with the ability to survive food limitation (improved foraging efficiency via ability to locate and then consume resources more efficiently; Beauchamp 1998, Beauchamp 2010); and should therefore be negatively correlated with migration distance (food limitation). Intraspecific nonbreeding group size during the stationary, nonbreeding period could be positively correlated with the ability to thermoregulate (if flocking species also roost communally and have more stored fat that improves ability to thermoregulate at night; group size may also reduce the need to thermoregulate via group huddling but may not affect ability to thermoregulate per se; Beauchamp 1999, Hay 1983), ability to fast between meals (if foraging in groups allows for larger fat reserves or roosting in groups improves overnight fasting ability because fat stores are metabolized slower; group size

may also reduce fasting durations via improved foraging efficiency but may not affect ability to fast per se), or ability to compete for resources (if larger groups have a competitive foraging advantage); and could therefore be negatively correlated with migration distance (thermal tolerance, fasting endurance, social dominance). Intraspecific nonbreeding group size during the stationary, nonbreeding period could be positively correlated with flight efficiency (if species that winter in flocks also migrate in flock formations that improve flight efficiency; Weimerskirch *et al*. 2001); and could therefore be positively correlated with migration distance in species that migrate in tight, flock formations (flight efficiency). Intraspecific nonbreeding group size during the stationary, nonbreeding period should not be correlated with ability to switch diet or competition for breeding opportunities; and should therefore not be correlated with migration distance (dietary specialization, arrival time).

Polygamy: Polygamy should be positively associated with competition for breeding opportunities (because of increased reproductive asymmetry within polygamous species); and should therefore be negatively associated with migration distance (arrival time). Polygamy could be negatively associated with nonbreeding ability to compete for resources (if polygamous species have greater variation in individual dominance status which could pull the mean distance farther away from breeding areas if subordinates are forced to migrate even farther); and could therefore be positively correlated with migration distance (social dominance). Polygamy should not be associated with ability to thermoregulate, ability to survive food limitation, ability to fast between meals, ability to switch diet, or flight efficiency; and should therefore not be associated with migration distance (thermal tolerance, food limitation, fasting endurance, dietary specialization, flight efficiency).

Tarsus feathering: Extent of tarsus feathering should be positively correlated with the ability to thermoregulate (because tarsus feathering reduces body heat loss through legs or feet); and should therefore be negatively correlated with migration distance. Extent of tarsus feathering should not be correlated with ability to survive food limitation, ability to fast between meals, ability to switch diet, ability to compete for resources, competition for breeding opportunities, or flight efficiency; and should therefore not be correlated with migration distance (food limitation, fasting endurance, dietary specialization, social dominance, arrival time, flight efficiency).

Territoriality: Territoriality should be positively correlated with the nonbreeding ability to compete for resources (because territorial birds can preclude others from otherwise shared resources); and should therefore be negatively correlated with migration distance (food limitation, social dominance). Territoriality could be positively correlated with competition for breeding opportunities (if territorial species have increased competition for breeding opportunities); and could therefore be negatively correlated with migration distance (arrival time). Territoriality should not be correlated with ability to thermoregulate, ability to fast between meals, ability to switch diet, or flight efficiency; and should therefore not be correlated with migration distance (thermal tolerance, fasting endurance, dietary specialization, flight efficiency).

Body mass x diet breadth interaction: If diet breadth is narrow, then body size could be even more negatively correlated with migration distance (positive interaction) because broader diets could improve the fasting ability of smaller birds via greater overnight fat stores (implies that broad diet improves fasting ability of smaller birds more than larger birds; fasting endurance). If diet breadth is narrow, then body size could be even more negatively correlated with migration distance (positive interaction) because broader diets reduce fasting durations (but

not fasting ability per se) of smaller birds more than larger birds (food limitation). Diet breadth does not affect the ability to thermoregulate and should not influence the relationship between body size and migration distance (thermal tolerance). Even if a larger body size improves the ability to switch diets (because larger individuals can consume a larger range of food sizes), diet breadth should not influence the relationship between body size and migration distance because diet breadth should be the main determinant of variation in migration distance (dietary specialization). Diet breadth should not affect the nonbreeding ability to compete for resources (dominant/more competitive birds should eat whatever they want, irrespective of diet breadth) and should not influence the relationship between body size and migration distance (social dominance). Neither diet breadth nor body size should be correlated with competition for breeding opportunities (arrival time). Diet breadth should not be correlated with flight efficiency and should not influence the relationship between body size and migration distance (flight efficiency).

Body mass x nocturnality interaction: Being active at night (i.e., more nocturnal) may be a food limitation adaptation that reduces fasting time between meals (because individuals can forage at night instead of fasting), therefore diurnal species should be even more affected by variation in fasting ability (which should be primarily driven by body size) creating a more negative relationship between body size and migration distance among species that are diurnal during the nonbreeding season (fasting endurance, food limitation; should also make the prediction that smaller species are more likely to be nocturnal during the stationary, nonbreeding period). Being active at night (i.e., more nocturnal) may be a thermoregulatory adaptation that can mitigate a lower ability to thermoregulate (because individuals can forage/move at night and generate metabolic heat instead of roosting), therefore diurnal species should be even more

affected by variation in thermoregulatory ability (which should be primarily driven by body size) creating a more negative relationship between body size and migration distance among species that are diurnal during the nonbreeding season (thermal tolerance; should also make the prediction that smaller species are more likely to be nocturnal during the stationary, nonbreeding period). Being active at night (i.e., more nocturnal) may be a competition adaptation that can mitigate lower nonbreeding competitive abilities (because individuals that are active at night encounter reduced competition for resources) therefore diurnal species should be even more affected by variation in nonbreeding competitive ability (which should be primarily driven by body size) creating a more negative relationship between body size and migration distance among species that are diurnal during the nonbreeding season (social dominance, food limitation; should also make the prediction that smaller species are more likely to be nocturnal during the stationary, nonbreeding period). Degree of nocturnality should not influence the relationship between body size and migration distance because degree of nocturnality should not be related to the ability to switch diet (dietary specialization). Degree of nocturnality should not influence the relationship between body size and migration distance because body size should not affect competition for breeding opportunities (arrival time). Degree of nocturnality should not influence the relationship between body size and migration distance because degree of nocturnality during the stationary, nonbreeding season should not be related to flight efficiency during migration (flight efficiency).

Body mass x nonbreeding group size interaction: If group size is small, then body size could be even more negatively correlated with migration distance (positive interaction) because: 1) living in larger groups could improve the ability of smaller birds to thermoregulate via group huddling while roosting (Beauchamp 1999, Hay 1983; thermal tolerance); 2) foraging in larger

groups could lead to larger fat stores (via improved foraging efficiency; Beauchamp 1998, Beauchamp 2010) that improve fasting ability of smaller birds (via net larger fat reserves or reserves are burned through more slowly via communal roosting; fasting endurance); 3) foraging in larger groups could improve the competitive ability of smaller birds relative to other groups or solitary foragers (social dominance); 4) foraging in larger groups leads to improved foraging efficiency resulting in reduced fasting durations (but not necessarily improved *ability* to fast between meals) in smaller birds (food limitation); or 5) larger birds that migrate in flock formations (and also winter in groups) benefit from improved flight efficiency during migration (flight efficiency). Intraspecific nonbreeding group size should not influence the relationship between body size and distance because group size should not be related to 1) the ability to switch diets (dietary specialization). Neither group size nor body mass should be related to competition for breeding opportunities (arrival time).

Body mass x hand-wing index/flight mode interaction: In species with reduced flight efficiency (flapping migrants or low hand-wing index), body size should be even more negatively correlated with migration distance (positive interaction) because larger body mass imposes significant constraints on flight energetics (e.g., body mass and migration speed are negatively correlated in species that use flapping flight; Hedenström & Alerstam 1998, Pennycuick 2008; flight efficiency). Neither wing shape nor flight mode should affect the ability to thermoregulate, the ability to survive food limitation, the ability to fast between meals, the ability to switch diet, or the nonbreeding ability to compete for resources; and should therefore not influence the relationship between body size and migration distance (thermal tolerance, food limitation, fasting endurance, dietary specialization, social dominance). Neither body size nor

wing shape/flight mode should be related to competition for breeding opportunities (arrival time).

2. Migration Distance

We used the following Web of Science and Google Scholar search string (developed using the "litsearcher" R package; Grames *et al.* 2019) which yielded 1460 and 4400 potential papers, respectively (last searched in July 2025): TS = ((bird* OR avian) AND ("satellite telemetry" OR "satellite tracking" OR "satellite transmitter" OR geolocator* OR "GPS tracking" OR "GPS telemetry" OR "GPS transmitter" OR "GSM tracking" OR "GSM telemetry" OR "GSM transmitter" OR "PTT tracking" OR "PTT telemetry" OR "PTT tracking" OR "GPS-GSM tracking" OR "GPS-GSM telemetry" OR "GPS-GSM transmitter") AND (migrat* OR "migrat* connectivity" OR "full annual cycle" OR "annual cycle")). We supplemented our search via Google Scholar Alerts for the following search strings which we used to find additional primary literature, theses, and dissertations that our Web of Science search may have missed: 1) (migration AND (avian* OR bird*)); 2) ("differential migration"); and 3) ("movement ecology"). We supplemented our search via Google Scholar alerts related to avian migration . Finally, we searched for additional studies within other comparative analyses (e.g., Wang et al. 2024; Watanabe 2016), meta-analyses (e.g., Trevail et al. 2023), or review papers (e.g., Cohen & Satterfield 2020) on avian migration. Mean migration distance was provided in the text of some studies, but we also estimated mean migration distance from maps or figures depicting breeding and nonbreeding locations, migration routes, or utilization distributions when this information was not available in the text (62% of studies).

3. Tarsus Feathering

We defined tarsus feathers as those feathers that insert directly into the ankle joint, tarsometatarsus, or digits, and did not include other feathers that may cover the tarsus but do not insert into them directly (e.g., vent or tibiotarsus feather). Tarsus feathering was scored as a continuous 0–10 metric as follows: 0 = no feathering on tarsus or ankle joint and 10 = tarsus and digits completely feathered (Figure S1).

4. Nest Substrate Availability

Nest substrate availability was a subjective score that took the following into account: nest substrate type (e.g., secondary cavity, primary cavity, tree, ground, etc.), diversity of nest substrates (if species use multiple types of substrates), habitat/substrate specificity within which nests were placed (e.g., emergent wetland vegetation, on cliffs behind waterfalls, etc.), and proportion of nest sites that are secondary cavities.

5. Secondary Cavity Use

Our definition of a secondary cavity included any site that functionally acted as a secondary cavity and was not constructed by the current occupants during the same breeding season. For example, a burrow excavated by a breeding pair in breeding season 1 was defined as a primary cavity, whereas that same previously excavated burrow used in breeding season 2 (even if used by the same pair) was defined as a secondary cavity. Secondary cavities included cavities in trees, underground (i.e., burrows), on cliffs (e.g., caves), in rock crevices, in human structures, and nest boxes.

6. Nonbreeding Daily Foraging Activity Pattern

We classified the daily temporal foraging activity patterns (i.e., degree of nocturnality) of species during the stationary, nonbreeding period as follows: 0 = strictly diurnal; 0.8 = diurnal and crepuscular; 1.4 = diurnal and sometimes nocturnal, but primarily diurnal; 5 = diurnal and

nocturnal; 8.6 = nocturnal and sometimes diurnal, but primarily nocturnal; 9.2 = nocturnal and crepuscular; 10 = strictly nocturnal. We scored species that were assumed to be primarily diurnal (e.g., many Passeriformes) or nocturnal as 0 or 10, respectively, but for which no specific information was located.

7. Intraspecific Nonbreeding Group Size

We used global eBird data (eBird Basic Dataset) to calculate a continuous, numerical estimate of intraspecific nonbreeding group size during the stationary nonbreeding period. We used data from "core" nonbreeding months (e.g., typically December and January for species breeding at northern arctic/temperate latitudes) for each species based on annual cycle phenology estimates (e.g., Billerman *et al.* 2022) to minimize the likelihood that we were including checklists during other portions of the annual cycle (e.g., breeding, migration). We further filtered checklists to minimize survey effort per checklist to maximize the likelihood that each checklist count contained as few intraspecific groups as possible. Ideally, we wanted each checklist to contain count data from only one intraspecific group, however eBird users do not differentiate among intraspecific group sizes within checklists. We filtered checklists to only include complete stationary, traveling, area, or pelagic checklists less than 30 minutes in duration and 0.5 km in length that recorded a numerical count value for the species of interest. We then calculated the mean checklist count for each species and used this as our estimate of relative intraspecific nonbreeding group size. We acknowledge that our filtering criteria likely selected for an unknown proportion of checklists where observer count totals included more than one intraspecific group. However, by standardizing our checklist filters we hoped to minimize this occurrence while also providing enough data to calculate an estimate of average nonbreeding group size for less abundant or more cryptic taxa. We further acknowledge that inclusion of

checklists where observer count totals included more than one intraspecific group may have biased mean group size higher (perhaps especially for more common and abundant taxa). We chose to use the mean, and not median, checklist count for each species because eBird checklists could be biased towards lower median counts (compared to the mean) if eBird users report a high volume of low count checklists (e.g., count = 1 individual) for vagrant records of the species of interest. However, we were interested in relative (not absolute) group size differences among species, and with no available estimates of mean group size for most species, we felt this exercise was an adequate method for calculating relative group size. For species with low nonbreeding checklist sample sizes (0–1 filtered checklists; n = 23 species), we used the mean count per checklist from all available checklists during the core nonbreeding months as our metric of nonbreeding group size. Most species (n = 15 of 23) suffering from low checklist sample sizes were pelagic during the nonbreeding months including 3 Charadriiformes and 12 Procellariiformes species.

To validate our methods for calculating intraspecific nonbreeding group size, we compared our group size estimates generated from eBird data to a binary flocking vs. solitary designation used in other analyses (e.g., Beauchamp 2022). Most of the binary species-level group size designations came from a single comparative database (Beauchamp 2022) that defined flocking species as those forming flocks (flock = groups with >2 adults) regularly, whereas non-flocking (solitary) species formed flocks irregularly or not at all. We also gathered additional binary group size designations from Birds of the World species accounts (Billerman et al. 2022). We used logistic regression to explain binary group size (0 = solitary; 1 = flocking) with our log transformed and standardized estimate of intraspecific nonbreeding group size.

8. Diet Breadth and Seasonal Food Availability

First, the percent composition of a species diet across 10 diet types was used to calculate a novel metric of diet breadth (EltonTraits 1.0; Wilman et al. 2014). We first multiplied each of the 10 diet composition categories by itself, took the summation of those 10 values, and then divided by the total sum of all diet category percentages (100%). The resulting values ranged from 16 (very diverse diet) to 100 (diet composed entirely of a single category). We then took the reciprocal and multiplied by 100 to generate a diet breadth score that had the potential to range from 1 – 10: 1 being a highly specialized diet (100% composition from a single diet category) and 10 being a very broad diet (10% composition from each of the 10 diet categories). Diet breadth scores in our database ranged from 1–6.25. Second, we used the same percent diet composition values (EltonTraits 1.0; Wilman et al. 2014) to calculate a novel metric of seasonal food availability that we defined as the relative availability of food on the breeding grounds during the nonbreeding season. Three researchers independently scored the following seven diet types, irrespective of species, on a relative scale from 1 (lowest availability on the breeding grounds during the nonbreeding season) to 5 (highest availability on the breeding grounds during the nonbreeding season): invertebrates, vertebrates (we collapsed the following four vertebrate diet categories for this exercise: endotherms, ectotherms, fish, unknown), scavenge, fruit, nectar, seed, and other plant material. We then used average scores among all experts within each diet type (Table S3) and used scores combined with percent diet composition to calculate a single weighted mean for each species that represented seasonal food availability on a possible scale of 1 to 5. Within species, inter-expert agreement was high with an average correlation of 0.89 among seasonal food availability indices (range = 0.82 – 0.93; Table S4).

9. Polygamy

We classified species as either mostly monogamous (polygamy rate < 5%) or polygamous (polygamy rate ≥ 5%). We chose 5% polygamy as the threshold separating mostly monogamous from polygamous species to be consistent with previous research (Snyder & Creanza 2019). We defined polygamy as the percentage of sampled birds with >1 simultaneous social mates. We searched Birds of the World species accounts (Billerman *et al.* 2022) and comparative literature (e.g., Snyder & Creanza 2019) for information on species' mating system. We estimated or assumed the degree of polygamy for many species based on verbiage from species accounts because polygamy percentage data was unavailable or unknown. If specific polygamy rates were not available but some low-level of polygamy was confirmed or suspected (usually via anecdotal accounts of single instances of polygamy), we took a conservative approach and classified species as monogamous unless the species mating system was specifically referred to as polygamous (e.g., often, frequently, etc.) in which case we classified species as polygamous. If we found multiple polygamy percentages for a given species, the midpoint of the low and high values was used for classification. Lekking species were classified as polygamous because they typically lack strong sexual bonds but still show strong asymmetry in reproductive success (Emlen & Oring 1977). Our definition of polygamy included both polygynous and polyandrous species.

10. Territoriality

Territoriality categorical definitions (defined in Tobias et al. 2016): 1 = non-territorial: species that never defend territories or defend only very small areas around nest sites; 2 = seasonal or weak territoriality: species with broadly overlapping home ranges or that join mixed flocks with poorly defined spatial boundaries; and 3 = year-round territoriality: species that defend territories all year, including migrants that defend both breeding and nonbreeding season territories.

11. Reproductive Investment

We could not locate egg mass values for all bird species (missing for n=6 for which we had egg length or egg width info but not mass). To overcome this limitation, we constructed linear models using egg length or width to predict egg mass for species where we had complete information on egg mass, length, and width. We divided our data into 4 quantiles of egg mass for species with empirical egg mass data because of concerns over non-linear allometric relationships between egg mass and length or width. Egg mass quantiles were as follows: 1) 1.2 g – 8.0 g; 2) 8.1 g – 39.2 g; 3) 39.3 g – 77.0 g; and 4) 77.1 g – 490.0 g. We ran linear models within each quantile with egg length or width and quadratic terms to predict egg mass. We compared R-squared values between models with linear versus quadratic predictors and chose models with the highest R-squared values for our calculations of predicted egg mass. These modeled equations were then used to impute missing egg mass values for species with empirical egg length or width data (n=6). Finally, we averaged modeled egg mass predicted by egg length and width to compute a single modeled egg mass value. We averaged egg length and egg width predicted egg mass values because exploratory analyses indicated overall improved predicted egg mass when compared to empirical egg mass values. Modeled egg mass formulas are provided in Table S10.

Section S2. Supplemental Results

1. Geographic and Body Size Sample Bias

We performed non-parametric Mann-Whitney tests to determine if the mass and breeding latitude of tracked species in our migration database was different than untracked species. To determine the mass and breeding latitude of untracked species, we used the "Mass" and "Centroid.Latitude" values, respectively from Tobias et al. (2022). "Centroid.Latitude" was

calculated as the geometric center of a species breeding range. The breeding latitude of tracked species used in our analysis (n = 446; mean = 36.75 ºN; median = 46.02 ºN) was significantly greater than all other bird species (n = 9,548; mean = 1.45 ºN; median = -1.01 ºS; $p < 0.0001$). Body size of tracked species used in our analysis (n = 446; mean = 988 g; median = 318 g) was significantly greater than all other bird species (n = 9,548; mean = 235 g; median = 34 g; $p < 0.0001$).

2. *Intraspecific Nonbreeding Group Size*

We found that our intraspecific measure of nonbreeding group size calculated from eBird data significantly explained binary group size (flocking vs. solitary; n = 379 species; ß= 2.27; SE = 0.221; $p < 0.0001$). The median intraspecific group size among solitary vs. flocking species was 2.0 (SD = 16) and 18 (SD = 93), respectively.

Table S1. Species traits and their predicted (deduced) relationships with migration distance based on underlying ecological mechanisms (in parentheses) of seven hypotheses explaining interspecific variation in migration distance.

	Deduced migration distance predictions from seven hypotheses						
Species trait or interaction	Thermal tolerance (nonbreeding ability to thermoregulate)	Food limitation (nonbreeding ability to survive food limitation)	Fasting endurance (nonbreeding ability to fast between meals)	Diet specialization (nonbreeding ability to switch diet)	Social dominance (nonbreeding ability to compete for resources)	Arrival time (competition for breeding opportunities)	Flight efficiency (mortality risk and energy efficiency during migration)
Bill length	positive[1]				sometimes negative[2]		sometimes negative[2]
Body mass	***negative***	***negative***	***negative***	sometimes negative[3]	***negative***		***sometimes negative[4]***
Diet breadth		negative		negative			sometimes positive[5]
Seasonal food availability		***negative***					
Flight mode							***positive***
Hand-wing index							***positive***
Nest substrate availability						positive	
Secondary cavity use	sometimes negative[6]					negative	
Nocturnality		negative[7]				sometimes positive[7]	
Nonbreeding group size	sometimes negative[8]	negative	sometimes negative[8]		sometimes negative[8]		sometimes positive[8]

Polygamy					sometimes positive[9]	negative[9]	
Tarsus feathering	negative						
Territoriality		negative			negative	sometimes negative	
Body mass x Diet breadth		sometimes positive[10]	sometimes positive[10]				
Body mass x Nocturnality	sometimes positive[11]	positive	sometimes positive[11]		sometimes positive[11]		
Body mass x Nonbreeding group size	***positive[12]***	***positive***	***positive[12]***		***sometimes positive[12]***		***sometimes positive[13]***
Body mass x Hand-wing index							positive[14]
Body mass x Flight mode							***positive[14]***

Note: Blank cells indicate a hypothesis makes no directional prediction. A priori predictions that were supported by our phylogenetic comparative analyses (i.e., 95% credible intervals from parameter estimates do not overlap zero) are italicized and bolded. Refer to Supplemental Methods for complete explanation and justification of all predictions.

[1] Allen's Rule (Symonds & Tattersall 2010); [2] If long bills increase competitive ability or drag in flight, respectively; [3] If diet breadth not accounted for; [4] In flapping species; [5] If broad diet improves migratory refueling; [6] If cavities used for winter roosting; [7] If being nocturnal decreases food limitation or competition for breeding, respectively; [8] If flocking species roost communally, store more fat, gain a competitive foraging advantage, or migrate in flocks, respectively; [9] If polygamous species have greater variation in dominance or reproductive asymmetry, respectively; [10] If broader diet improves fasting ability more in smaller species; [11] If diurnal species more affected by thermoregulatory ability, fasting ability, or competitive ability because not active at night, do not forage at night, or greater amount of diurnal competition, respectively; [12] If solitary species more affected by thermoregulatory ability, fasting ability, or competitive ability because

no communal roosting, reduced fat stores, or larger groups more dominant, respectively; [13] If larger species benefit more from migrating in flocks; [14] Body size even more negatively related to distance when hand-wing index low or in flapping species, respectively.

Table S2. Summary of predictor and response variables used in migration distance analyses. For ease of interpretation, we present summary statistics from raw data (e.g., before transformation or standardization).

Variable	Type	Description	Summary
Migration distance	Response	Mean population-level migration distance in kilometers	Mean = 3,477; Median = 2,661; Range = 103 - 16,225
Absolute breeding latitude	Predictor: Control	Absolute value of population breeding latitude	Mean = 47.3; Median = 47.7; Range = 3.49 - 81.6
Body mass	Predictor	Mean species body mass in grams	Mean = 988.1; Median = 318.0; Range = 8.20 - 11,071
Bill length	Predictor	Mean species culmen length in millimeters	Mean = 49.5; Median = 37.5; Range = 8.0 - 359
Hand-wing index	Predictor	100 x Kipp's distance/wing length	Mean = 44.6; Median = 45.3; Range = 15.5 - 73.0
Tarsus feathering	Predictor	Extent of tarsus feathering on a scale of 0 (no tarsus feathering) to 10 (tarsus and digits completely feathered)	Mean = 1.24; Median = 0.00; Range = 0 - 10; NA = 1
Nest substrate availability	Predictor	Nesting substrate availability scored on a scale of 1 (low availability) to 5 (high availability)	Mean = 3.19; Median = 4.00; Range = 1.00 - 5.00; NA = 10
Secondary cavity use	Predictor	Proportion of nesting sites that are secondary cavities	Mean = 0.18; Median = 0.00; Range = 0.00 - 1.00; NA = 5
Polygamous	Predictor	Species with a polygamous mating system (≥ 5% polygamy) or not (< 5% polygamy)	No = 369; Yes = 41; NA = 36
Nocturnality	Predictor	Degree of nocturnality during the stationary, nonbreeding period on a scale of 0 (strictly diurnal) to 10 (strictly nocturnal)	Mean = 1.58; Median = 0.00; Range = 0 - 10; NA = 45
Group size	Predictor	Mean intraspecific group size during the stationary nonbreeding season	Mean = 23.8; Median = 5.00; Range = 1 - 826
Diet breadth	Predictor	Diet breadth on a possible scale of 1 (highly specialized diet) to 10 (highly diversified diet)	Mean = 1.92; Median = 1.72; Range = 1.00 - 6.25
Seasonal food availability	Predictor	The availability of food on the breeding grounds during the nonbreeding period based on diet type on a scale of 1 (low availability) to 5 (high availability)	Mean = 2.79; Median = 2.73; Range = 1.07 - 5.00
Territoriality	Predictor	Degree of territoriality from non-territorial to year-round territorial	Non-territorial = 247; Seasonal or weakly = 180; Year-round = 10
Flight mode	Predictor	Flight mode used during migration	Flapping = 261; Flap/soar mix = 128; Soaring = 48; NA = 9
Survival	Life History	Adult annual survival estimate	Mean = 0.74; Median = 0.78; Range = 0.33 - 0.98

Minimum reproductive investment	Life History	(egg mass/body mass) * clutch size	Mean = 0.34; Median = 0.29; Range = 0.03 - 1.26; NA = 8
Maximum reproductive investment	Life History	[(egg mass/body mass) * clutch size] * max. number of successful broods per breeding season	Mean = 0.6; Median = 0.32; Range = 0.02 – 3.13; NA = 24

Table S3. Seasonal food availability scores for seven diet types averaged among three independent experts. The average score of each diet type was used to calculate each species index of seasonal food availability.

**Diet Type**	**Diet Score**			
	**Expert 1**	**Expert 2**	**Expert 3**	**Average**
Invertebrates	1	2	2	1.67
Vertebrates	4	4	5	4.33
Scavenger	5	5	5	5.00
Fruit	2	3	1.5	2.17
Nectar	1	1	1	1.00
Seeds	3	5	3	3.67
Plants	3	3	2.5	2.83

Table S4. Within species correlations of seasonal food availability indices based on expert scoring from three independent researchers combined with standardized foraging data (Wilman et al. 2014).

	Seasonal food availability: Expert 2	Seasonal food availability: Expert 3
Seasonal food availability: Expert 1	0.934	0.919
Seasonal food availability: Expert 2		0.819

Table S5. Individual model parameter estimates for each migration distance predictor(s) associated with a single prediction. All models include phylogenetic relatedness (Lambda) from a single, randomly selected phylogenetic tree as a random effect. Retained parameters of interest (marked "Y") were incorporated into a single model used for further analysis in Table S5.

Model	Parameter of Interest	Parameter estimate w. 95% credible intervals	Lambda	Number of Species	Retained for Table S5?	Comments
Distance ~ Latitude	Latitude	0.40 (0.31, 0.49)	0.66	446	Y	
Distance ~ Latitude + Bill Length	Bill Length	-0.03 (-0.18, 0.12)	0.66	446	N	
Distance ~ Latitude + Body Mass	Body Mass	-0.44 (-0.62, -0.27)	0.63	446	Y	
Distance ~ Latitude + Diet Breadth	Diet Breadth	-0.07 (-0.17, 0.03)	0.64	446	N	
Distance ~ Latitude + Body Mass + Diet Breadth	Diet Breadth	-0.05 (-0.15, 0.05)	0.61	446	N	Testing dietary specialization hypothesis
Distance ~ Latitude + Seasonal Food Availability	Seasonal Food Availability	-0.29 (-0.42, -0.16)	0.61	446	Y	
Distance ~ Latitude + Flight Mode	Flight Mode	flap/soar: 0.29 (-0.03, 0.61); soar: 0.47 (-0.07, 1.00)	0.64	437	N	reference category: obligate flapping
Distance ~ Latitude + Hand-wing Index	Hand-wing Index	0.43 (0.27, 0.59)	0.60	446	Y	
Distance ~ Latitude + Nest Substrate Availability	Nest Substrate Availability	-0.04 (-0.13, 0.06)	0.63	436	N	
Distance ~ Latitude + Secondary Cavity Use	Secondary Cavity Use	0.02 (-0.08, 0.12)	0.65	441	N	
Distance ~ Latitude + Nocturnality	Nocturnality	-0.07 (-0.17, 0.05)	0.55	401	N	
Distance ~ Latitude + Nonbreeding Group Size	Nonbreeding Group Size	-0.01 (-0.11, 0.09)	0.66	446	N	
Distance ~ Latitude + Polygamy	Polygamy	-0.21 (-0.51, 0.09)	0.66	410	N	
Distance ~ Latitude + Tarsus Feathering	Tarsus Feathering	-0.12 (-0.30, 0.05)	0.68	445	N	
Distance ~ Latitude + Territoriality	Territoriality	seasonal: -0.05 (-0.29, 0.19); year-round: -0.46 (-1.01, -0.12)	0.64	446	Y	reference category: non-territorial

Table S6. Individual model parameter estimates for each migration distance predictor added to our retained main effects model from Table S4. All models include phylogenetic relatedness (Lambda) from a single, randomly selected phylogenetic tree as a random effect. Additional retained parameters of interest (marked "Y") were incorporated into a top main effects model for further analysis in Table S6.

Model	Parameter of Interest	Parameter estimate w. 95% credible intervals	Lambda	Number of Species	Retained for Table S6?	Notes
Model 1: Latitude + Body Mass + Hand-wing Index + Seasonal Food Availability	NA	NA	0.53	446	Y	All parameters retained
Model 1 + Bill Length	Bill Length	0.04 (-0.10, 0.17)	0.52	446	N	
Model 1 + Diet Breadth	Diet Breadth	-0.02 (-0.11, 0.08)	0.52	446	N	
Model 1 + Flight Mode	Flight Mode	flap/soar: 0.22 (-0.08, 0.52); soar: 0.92 (0.42, 1.42)	0.41	437	Y	reference category: obligate flapping
Model 1 + Nest Substrate Availability	Nest Substrate Availability	-0.01 (-0.10, 0.08)	0.51	436	N	
Model 1 + Secondary Cavity Use	Secondary Cavity Use	-0.01 (-0.10, 0.08)	0.52	441	N	
Model 1 + Nocturnality	Nocturnality	-0.08 (-0.19, 0.02)	0.40	401	N	Parameter estimate only just includes zero
Model 1 + Nonbreeding Group Size	Nonbreeding Group Size	-0.04 (-0.14, 0.06)	0.51	446	N	
Model 1 + Polygamy	Polygamy	-0.10 (-0.39, 0.18)	0.55	410	N	
Model 1 + Tarsus Feathering	Tarsus Feathering	-0.09 (-0.24, 0.06)	0.54	445	N	
Model 1 + Territoriality	Territoriality	seasonal: 0.07 (-0.16, 0.30); year-round: -0.28 (-0.82, 0.26)	0.52	446	N	

Table S7. Individual model parameter estimates for each migration distance interaction term added to our top main effect model retained from Table S5. All models include phylogenetic relatedness (Lambda) from a single, randomly selected phylogenetic tree as a random effect. Models retained for main inference (marked "Y") include only parameter estimates with 95% credible intervals that exclude zero.

Model	Parameter of Interest	Parameter estimate w. 95% credible intervals	Lambda	Number of Species	Retained for Main Inference	Notes
Model 2: Latitude + Body Mass + Hand-wing Index + Flight Mode + Seasonal Food Availability	NA	NA	0.41	437	Y	Top main effects model: Fig. 3, Fig. S4
Model 2 + Body Mass*Nonbreeding Group Size	Body Mass*Nonbreeding Group Size	0.11 (0.01, 0.21)	0.38	437	Y	Fig. 4A
Model 2 + Body Mass*Flight Mode	Body Mass*Flight Mode	flap/soar: 0.33 (0.04, 0.63); soar: 0.50 (-0.07, 1.04)	0.38	437	Y	Fig. 4B
Model 2 + Body Mass*Hand-wing Index	Body Mass*Hand-wing Index	0.11 (-0.02, 0.24)	0.39	437	N	Parameter estimate only just includes zero
Model 2 + Body Mass*Diet Breadth	Body Mass*Diet Breadth	0.04 (-0.05, 0.14)	0.41	437	N	
Model 2 + Body Mass*Nocturnality	Body Mass*Nocturnality	-0.06 (-0.20, 0.07)	0.26	395	N	

Table S8. Individual model parameter estimates for each migration distance life history predictor. All models include phylogenetic relatedness (Lambda) from a single, randomly selected phylogenetic tree as a random effect.

Model	Parameter of Interest	Parameter estimate w. 95% credible intervals	Lambda	Number of Species
Distance ~ Latitude + Survival (empirical & modeled)	Survival	-0.03 (-0.14, 0.08)	0.66	446
Distance ~ Latitude + Survival (empirical only)	Survival	0.01 (-0.10, 0.13)	0.61	323
Distance ~ Latitude + Min. Reproductive Investment	Min. Reproductive Investment	0.09 (-0.06, 0.23)	0.65	438
Distance ~ Latitude + Max. Reproductive Investment	Max. Reproductive Investment	-0.13 (-0.26, -0.01)	0.64	422

Table S9. Correlations among migration distance predictor variables included in top models.

	Mass	Hand-wing Index	Flight Mode	Seasonal Food Availability	Intraspecific Nonbreeding Group Size
Latitude	-0.001	0.007	0.012	-0.002	0.011
Mass		0.127	0.226	0.356	0.092
Hand-wing Index			0.220	0.018	0.147
Flight Mode				0.260	0.013
Seasonal Food Availability					-0.002

Table S10. Generalized variance inflation factors (GVIF) for migration distance predictor variables included in top models.

Predictor	GVIF (PGLS)	GVIF (GLS)
Latitude	1.02	1.06
Mass	1.44	2.20
Hand-wing Index	1.27	1.73
Flight Mode	1.36	2.11
Seasonal Food Availability	1.24	1.85
Intraspecific Nonbreeding Group Size	1.13	1.45

Note: Calculating VIFs from PGLMMs within brms was not possible so we calculated GVIFs using the vif function within the 'car' package (Fox & Weisberg 2019) by fitting top models using phylogenetic generalized least squares (PGLS) and generalized least squares (GLS) via the gls function within the 'nlme' package (Pinheiro et al. 2024).

Table S11. Empirical linear or quadratic relationships between egg length or egg width and egg mass (Prediction Formulas) used to impute missing egg mass values. We used empirical egg length or empirical egg width to predict empirical egg mass across four quantiles of egg mass values (Egg Mass Range). Sample sizes (Number of Species) used to generate prediction formulas are shown. We show adjusted-R^2 values from linear models comparing empirical egg mass with modeled egg mass (modeled egg mass averaged from egg length and egg width prediction formulas).

Egg Mass Range	Number of Species (n)	Prediction Formula: Egg Mass ~ Egg Length	Prediction Formula: Egg Mass ~ Egg Width	Adjusted-R^2 from Empirical~Modeled Egg Mass
1.2 - 8.0 g	100	4.203563 - 0.485564*(egg length) + 0.019458*(egg length^2)	2.23235 - 0.46130*(egg width) + 0.03159*(egg width^2)	0.9798
8.1 - 39.2 g	99	-30.75799 + 1.26554*(egg length)	-39.30193 + 1.99345*(egg width)	0.9382
39.3 - 77.0 g	99	-44.3572 + 1.6995*(egg length)	-68.5892 + 2.9457*(egg width)	0.8576
77.1 - 490 g	99	284.98245 - 7.79778*(egg length) + 0.07251*(egg length^2)	354.68091 - 15.60194*(egg width) + 0.20975*(egg width^2)	0.9830

Figure S1. Variation in avian tarsus feathering. Examples of tarsus (i.e., tarsometatarsus and digits) feather scores: (A) 0 – no feathering on tarsus (*Charadrius semipalmatus*); (B) 1.6 – feathering only extends to cover the intertarsal joint (*Setophaga kirtlandii*); (C) 3 – feathering extends beyond the intertarsal joint but covers less than half of the tarsometatarsus (*Buteo swainsoni*); (D) 5.9 – feathering extends beyond the intertarsal joint but covers more than half of the tarsometatarsus (*Haliaeetus leucocephalus*); (E) 7.6 – feathering covers the entire tarsometatarsus but digits are unfeathered (*Buteo lagopus*); and (F) 10 – feathering completely covers the tarsometatarsus and digits (*Aegolius acadicus*). All example species were included in this paper except for *Aegolius acadicus*. All photos taken by Neil Paprocki.

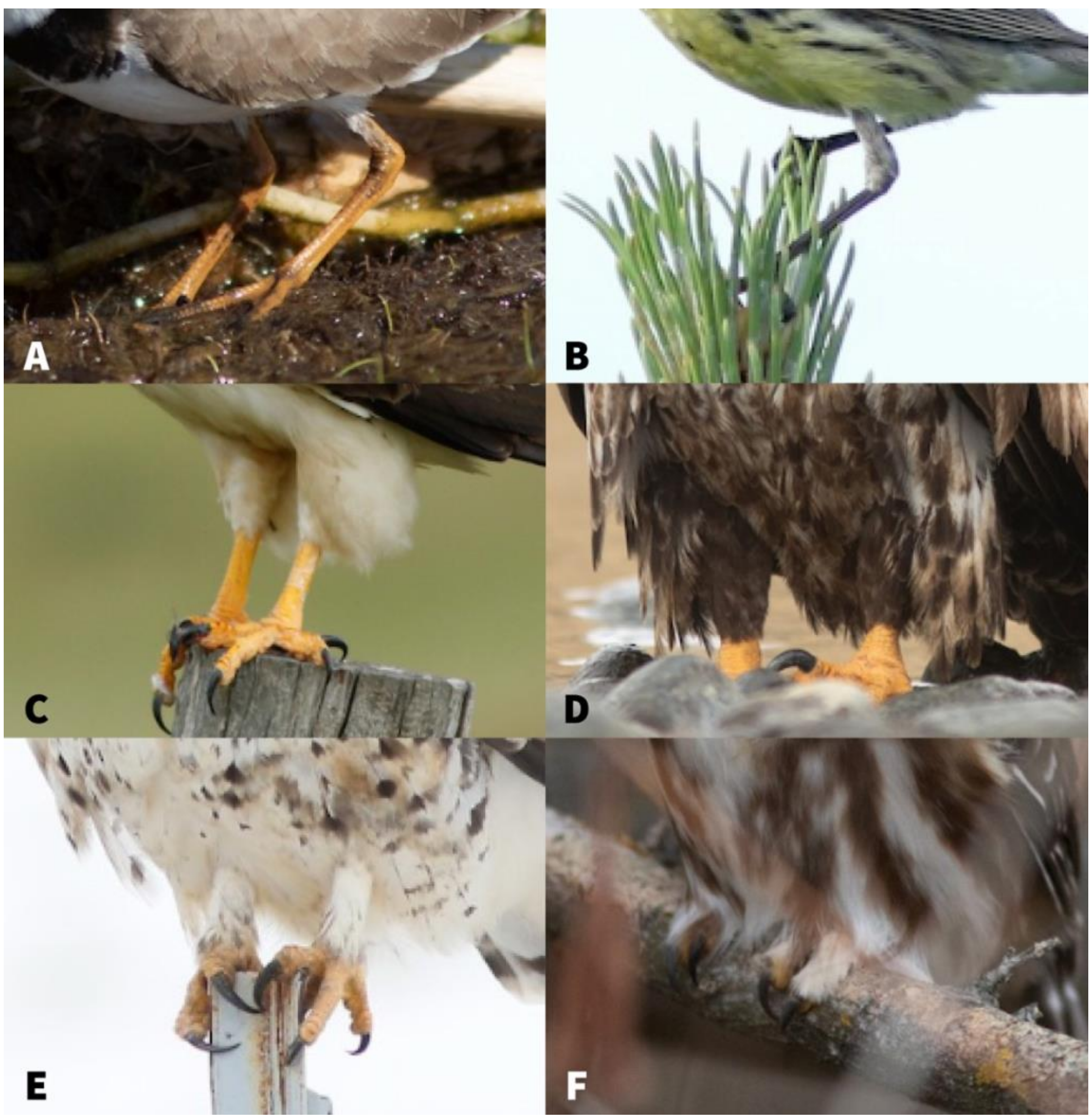

Figure S2. Relationship between log transformed body mass and either A) untransformed bill length or B) log transformed bill length.

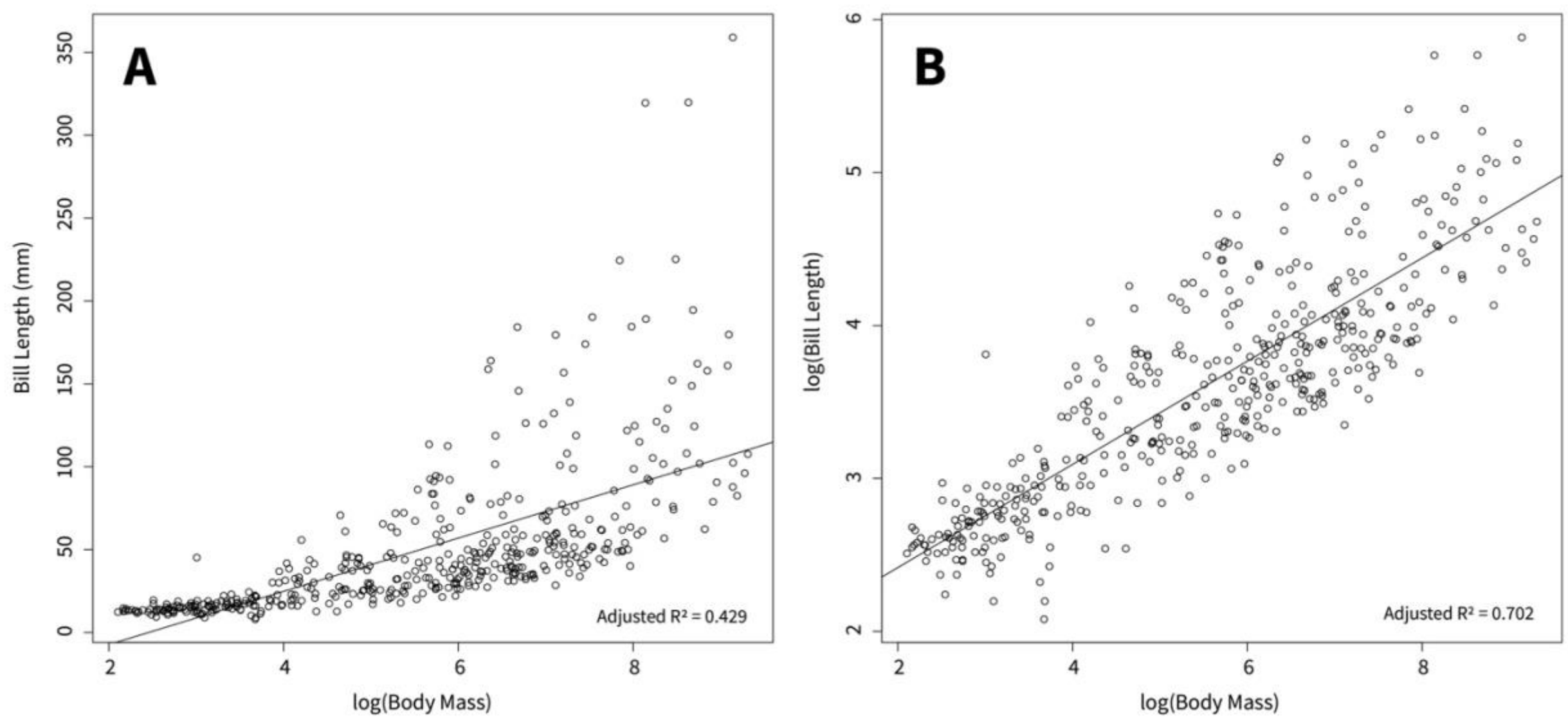

Figure S3. Relationship between log transformed body mass and either A) untransformed adult annual survival or B) log transformed adult annual survival.

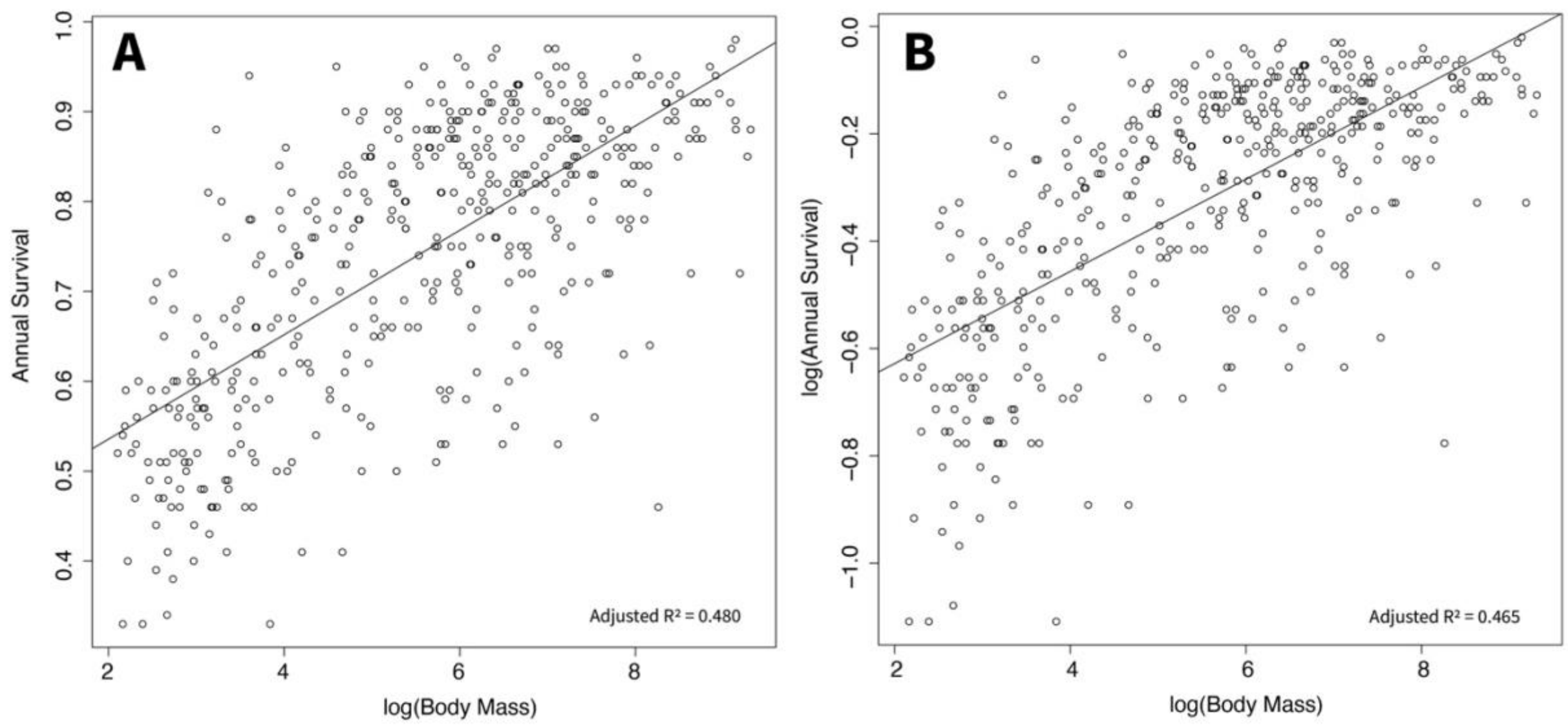

Figure S4. Relationships between log transformed body mass and A) untransformed minimum reproductive investment; B) log transformed minimum reproductive investment; C) untransformed maximum reproductive investment; and D) log transformed maximum reproductive investment.

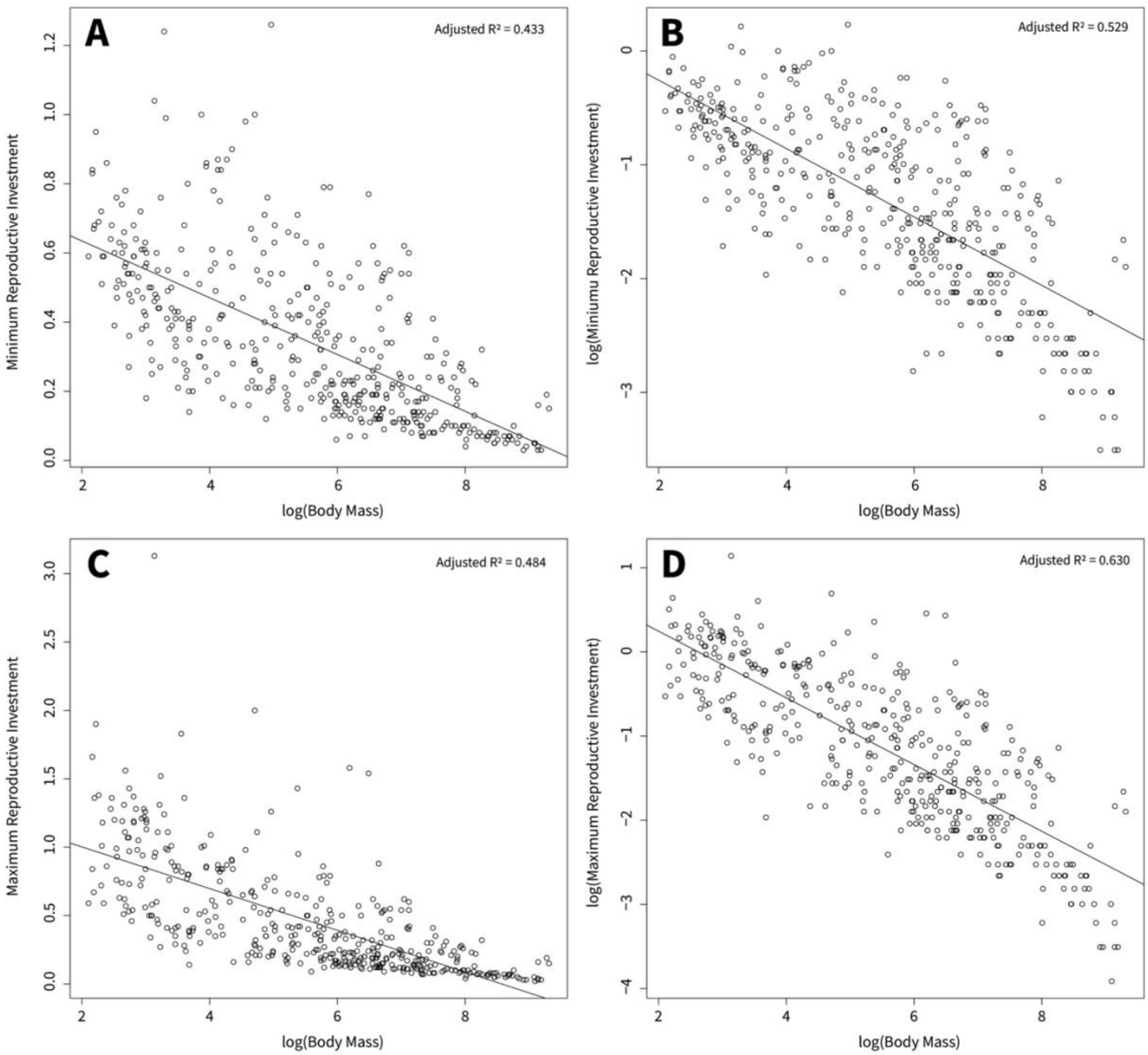

Figure S5. Absolute breeding latitude is positively correlated with migration distance among bird species. Trend lines and shaded regions (representing 95% credible intervals) are from a Bayesian phylogenetic generalized linear mixed model predicting log-transformed and standardized migration distance including body mass, hand-wing index, seasonal food availability, and flight mode as predictor variables (top distance main effects model). Raw data plotted as gray points (1 per species) for reference but does not account for the conditional effects of other variables included in models.

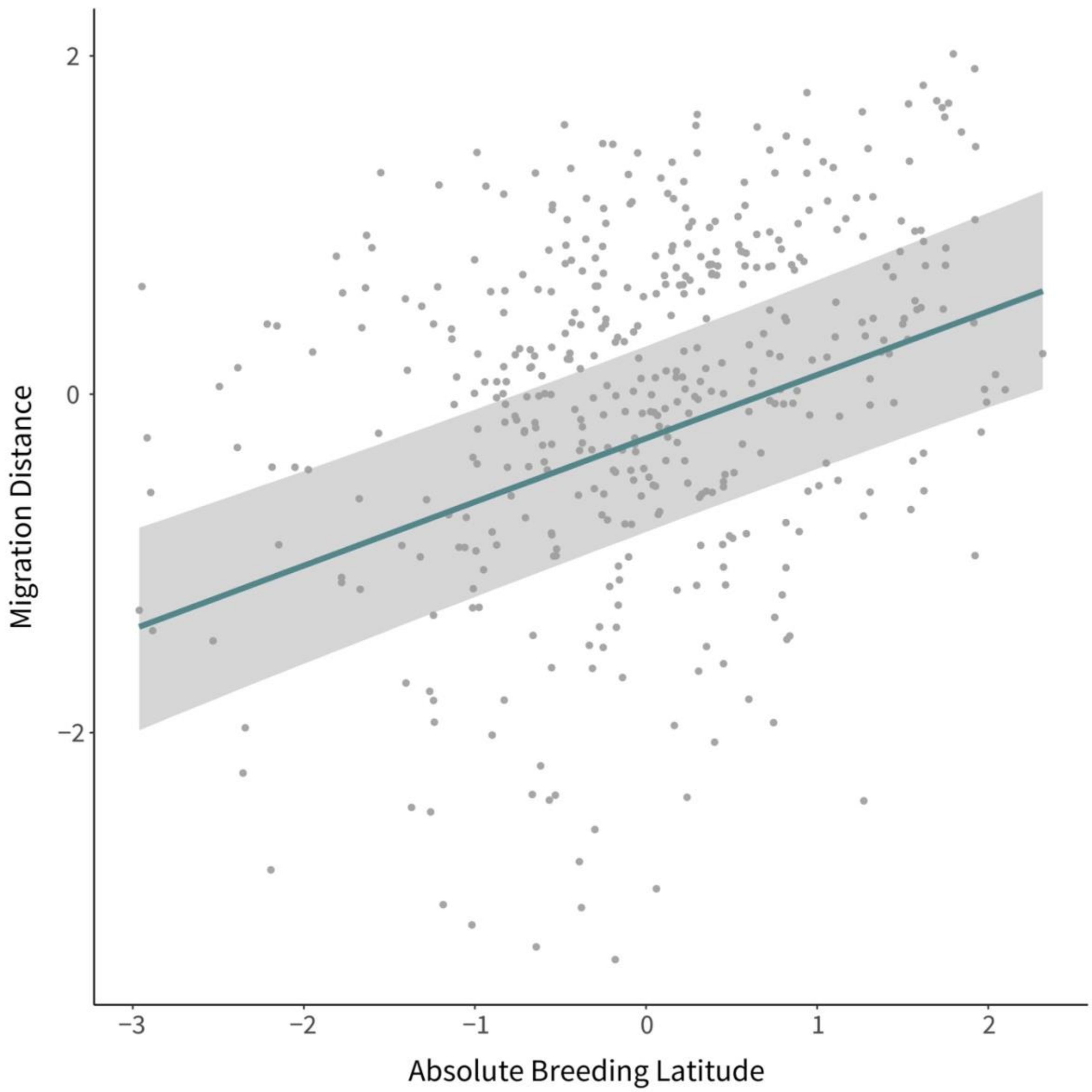

References

Beauchamp, G. (1998) The effect of group size on mean food intake rate in birds. *Biological Reviews*, 73, 449–472.

Beauchamp, G. (1999) The evolution of communal roosting in birds: origin and secondary losses. *Behavioral Ecology*, 10, 675–687.

Beauchamp, G. (2010) Relaxed predation risk reduces but does not eliminate sociality in birds. *Biology Letters*, 6, 472–474.

Beauchamp, G. (2022) Flocking in birds is associated with diet, foraging substrate, timing of activity, and life history. Behavioral Ecology and Sociobiology, 76, 74.

Billerman, S.M., Keeney, B.K., Rodewald, P.G. & Schulenberg, T.S. (Eds.) (2022) *Birds of the World*. Cornell Laboratory of Ornithology, Ithaca, NY. Available at: https://birdsoftheworld.org

Boyle, W.A., Conway, C.J. & Bronstein, J.L. (2011) Why do some, but not all, tropical birds migrate? A comparative study of diet breadth and fruit preference. *Evolutionary Ecology*, 25, 219–236.

Calder, W.A. (1974) Consequences of body size for avian energetics. In: Paynter, R.A. (Ed.) *Avian Energetics*. Cambridge, MA: Nuttall Ornithological Club, pp. 86–144.

Cohen, E.B. & Satterfield, D.A. (2020) 'Chancing on a spectacle:' co-occurring animal migrations and interspecific interactions. *Ecography*, 43, 1–15.

eBird Basic Dataset. (2024) Version: EBD_relMay-2024. Cornell Lab of Ornithology, Ithaca, New York. Last accessed May 2024.

Elner, R.W. & Seaman, D.A. (2003) Calidrid conservation: unrequited needs. *Bulletin-Wader Study Group*, 100, 30–34.

Emlen, S.T. & Oring, L.W. (1977) Ecology, sexual selection, and the evolution of mating systems. *Science*, 197, 215–223.

Fox, J. & Weisberg, S. (2019) *An R companion to applied regression, 3rd edn*. Thousand Oaks, CA: Sage publications.

Gauthreaux, S.A. (1978) The ecological significance of behavioural dominance. In: Bateson, P.P.G. & Klopfer, P.H. (Eds.) *Perspectives in Ethology, vol. 3*. New York, NY: Plenum Press, pp. 17–54.

Grames, E.M., Stillman, A.N. & Tingley, M.W. (2019) An automated approach to identifying search terms for systematic reviews using keyword co-occurrence networks. *Methods in Ecology and Evolution*, 10, 1645–1654.

Hay, D.B. (1983) Physiological and behavioral ecology of communally roosting Pygmy Nuthatch (*Sitta pygmaea*). Phd Thesis, Northern Arizona University, Flagstaff, AZ.

Hedenström, A. & Alerstam, T. (1998) How fast can birds migrate? *Journal of Avian Biology*, 29, 424–432.

James, F.C. (1970) Geographic size variation in birds and its relationship to climate. *Ecology*, 51, 365–390.

Ketterson, E.D. & Nolan Jr., V. (1976) Geographic variation and its climatic correlates in the sex ratio of eastern-wintering dark-eyed juncos (*Junco hyemalis hyemalis*). *Ecology*, 57, 679–693.

Leighton, G.M., Lamour, D., Malcolm, K. & Miller, E.T. (2022) Both morphological and behavioral traits predict interspecific social dominance in birds. *Journal of Ornithology*, 164, 163–169.

Lockwood, R., Swaddle, J.P. & Rayner, J.M.V. (1998) Avian wingtip shape reconsidered: wingtip shape indices and morphological adaptations to migration. *Journal of Avian Biology*, 29, 273–292.

McNab, B.K. (1971) On the ecological significance of Bergmann's Rule. *Ecology*, 52, 845–854.

Paprocki, N. & Conway, C.J. (2025) The underlying causes of differential migration: assumptions, hypotheses, and predictions. *Biological Review*s, 100, 764–789.

Pennycuick, C.J. (2008) *Modelling the flying bird*. Amsterdam: Elsevier.

Pinheiro, J., Bates, D. & R Core Team. (2024) nlme: linear and nonlinear mixed effects models. R package version 3.1-166, https://CRAN.R-project.org/package=nlme.

Schoener, T.W. (1967) The ecological significant of sexual dimorphism in size in the lizard *Anolis conspersus*. *Science*, 155, 474–477.

Sheard, C., Neate-Clegg, M.H.C., Alioravainen, N., Jones, S.E.I., Vincent, C., Macgregor, H.E.A., et al. (2020) Ecological drivers of global gradients in avian dispersal inferred from wing morphology. *Nature Communications*, 11, 2463.

Snyder, K.T. & Creanza, N. (2019) Polygyny is linked to accelerated birdsong evolution but not to larger song repertoires. *Nature Communications*, 10, 884.

Symonds, M.R.E. & Tattersall, G.J. (2010) Geographical variation in bill size across bird species provides evidence for Allen's Rule. *The American Naturalist*, 176, 188–197.

Tobias, J.A., Sheard, C., Seddon, N., Meade, A., Cotton, A.J. & Nakagawa, S. (2016) Territoriality, social bonds, and the evolution of communal signaling in birds. *Frontiers in Ecology and Evolution*, 4, 74.

Tobias, J.A., Sheard, C., Pigot, A.L., Devenish, A.J.M., Yang, J., Sayol, F. et al. (2022) AVONET: morphological, ecological and geographical data for all birds. *Ecology Letters*, 25, 581–597.

Trevail, A.M., Nicoll, M.A.C., Freeman, R. Le Corre, M., Schwarz, J., Jaeger, A., Bretagnolle, V., et al. (202 Tracking seabird migration in the tropical Indian Ocean reveals basin-scale conservation need. *Current Biology*, 33, 1–10.

Wang, X., Somveille, M., Dokter, A.M., Cao, W., Cheng, C., Liu, J. & Ma, Z. (2024) Macro-scale relationship between body mass and timing of bird migration. *Nature Communications*, 15, 4111.

Watanabe, Y.Y. (2016) Flight mode affects allometry of migration range in birds. *Ecology Letters*, 19, 907–914.

Weimerskirch, H., Martin, J., Clerquin, Y., Alexandre, P. & Jiraskova, S. (2001) Energy saving in flight formation. *Nature*, 413, 697–698.

Wilman, H., Belmaker, J., Simpson, J., de la Rosa, C., Rivadeneira, M.M. & Jetz, W. (2014) EltonTraits 1.0: species-level foraging attributes of the world's birds and mammals. *Ecology*, 95, 2027.
